# Comprehensive analysis of complete chloroplast genome and phylogenetic aspects of ten *Ficus* species

**DOI:** 10.1186/s12870-022-03643-4

**Published:** 2022-05-23

**Authors:** Yuying Huang, Jing Li, Zerui Yang, Wenli An, Chunzhu Xie, Shanshan Liu, Xiasheng Zheng

**Affiliations:** 1 Institute of Medicinal Plant Physiology and Ecology, School of Pharmaceutical Sciences, Guangzhou University of Chinese Medicine, 232th Waihuangdong Road, Higher Education Mega Center, Panyu District, Guangzhou, Guangdong China; 2 Traditional Chinese Medicine Gynecology Laboratory in Lingnan Medical Research Center, Guangzhou University of Chinese Medicine, Guangzhou, 510410 China

**Keywords:** *Ficus*, Chloroplast genome, Genome structure, Molecular markers, Phylogenetic analysis

## Abstract

**Background:**

The large genus *Ficus* comprises approximately 800 species, most of which possess high ornamental and ecological values. However, its evolutionary history remains largely unknown. Plastome (chloroplast genome) analysis had become an essential tool for species identification and for unveiling evolutionary relationships between species, genus and other rank groups. In this work we present the plastomes of ten *Ficus* species.

**Results:**

The complete chloroplast (CP) genomes of eleven *Ficus* specimens belonging to ten species were determined and analysed. The full length of the *Ficus* plastome was nearly 160 kbp with a similar overall GC content, ranging from 35.88 to 36.02%. A total of 114 unique genes, distributed in 80 protein-coding genes, 30 tRNAs, and 4 rRNAs, were annotated in each of the *Ficus* CP genome. In addition, these CP genomes showed variation in their inverted repeat regions (IR). Tandem repeats and mononucleotide simple sequence repeat (SSR) are widely distributed across the *Ficus* CP genome. Comparative genome analysis showed low sequence variability. In addition, eight variable regions to be used as potential molecular markers were proposed for future *Ficus* species identification. According to the phylogenetic analysis, these ten *Ficus* species were clustered together and further divided into three clades based on different subgenera. Simultaneously, it also showed the relatedness between *Ficus* and *Morus*.

**Conclusion:**

The chloroplast genome structure of 10 *Ficus* species was similar to that of other angiosperms, with a typical four-part structure. Chloroplast genome sizes vary slightly due to expansion and contraction of the IR region. And the variation of noncoding regions of the chloroplast genome is larger than that of coding regions. Phylogenetic analysis showed that these eleven sampled CP genomes were divided into three clades, clustered with species from subgenus *Urostigma*, *Sycomorus*, and *Ficus*, respectively. These results support the Berg classification system, in which the subgenus *Ficus* was further decomposed into the subgenus *Sycomorus*. In general, the sequencing and analysis of *Ficus* plastomes, especially the ones of species with no or limited sequences available yet, contribute to the study of genetic diversity and species evolution of *Ficus*, while providing useful information for taxonomic and phylogenetic studies of *Ficus*.

**Supplementary Information:**

The online version contains supplementary material available at 10.1186/s12870-022-03643-4.

## Background

The genus *Ficus,* which composes one of the 50 largest genera of angiosperms with approximately 800 species, is widely distributed in the tropical and semi-tropical temperate zones [1, 2]. Plants in this genus play a vital role in the ecosystem and are considered to be key species in tropical rainforests, because they serve as an extremely important source of food for frugivores species throughout the year [3, 4]. In addition, many *Ficu*s species are traditionally used as sources of medicines and food, as ornamental resources, religious plants, lac hosts, fodder, fuel, hedges, or enclosures by humans [5, 6]. Over the past decades, extensive investigation on pharmacological studies has elucidated the medicinal properties of *Ficus* species, including antioxidant [7], anti-microbial [8], anti-cancer [9], anti-inflammatory [10] and anti-diabetic [11] properties. Therefore, the superposition of dietary and medicinal values endows many *Ficus* species with high research value, especially to Chinese people [12].

*Ficus* (Moraceae) is a key group of tropical and subtropical plants with extremely important ecological significance, with the phylogenetic relationships of this group under controversy [13]. In 1965, Corner published a revised and more comprehensive classification system of *Ficus*, in which the *Ficus* genus was divided into four subgenera based on morphological characteristics including male flowers, female flowers, fruit characters, and some anatomical characters of leaves (such as the distribution of camphor), namely subgenus *Urostigma*, subgenus *Pharmacosycea*, subgenus *Ficus* and subgenus *Sycomorus* [14]. But this classification system has been questioned by Ramirez [15] and Berg [1, 2]. Hereafter, based on the morphologic study by Corner and the molecular systematics study by Weiblen [16], Berg added another two subgenera, namely *Sycidium* and *Synoecia*, in addition to the original 4 subgenera [2]. Although the classification system in those 6 subgenera has been accepted by most taxonomists, emphasis was laid on the *Ficus* genus, raising issues such as classification difficulties and incomplete collection of species, which renders this classification still unresolved.

With the advances of next-generation sequencing [17], the acquisition of whole genomes becomes easier than before. As an important organelle in plants, CP contains the whole enzymatic machinery, which is necessary for photosynthesis and plays a crucial role in carbon uptake [18]. Simultaneously, it possesses a small, highly conserved genome that takes the form of a circular double-stranded DNA molecule. In most angiosperms, the typical CP genome exhibits a conserved tetrad structure, formed by two IRs, one LSC region and one SSC region [19]. In general, the size of the CP genome ranges from 115 to 165 kb, owing to a contraction or expansion of the IR region. Additionally, the CP genome contains approximately 114 genes, among which there are ~ 80 protein-coding genes, 4 rRNA genes, and 30 tRNA genes [20, 21]. Even though the plant CP genome is evolutionarily conserved, it presents highly variable regions that some of them exhibit an accelerated evolution rate [22–24]. Based on these characteristics, the CP genome is often used for phylogenetic and evolutionary studies, and has been proved useful for screening species-specific genetic markers, i.e. DNA barcoding, SNPs, among others in recent years [25–28]. Therefore, we expect that plastome comparative genomics on more *Ficus* species might provide insights on *Ficus* taxonomic and phylogenetic concerns raised previously, and will allow the development of DNA barcodes for a reliable identification of *Ficus* species.

In this study, ten *Ficus* plastomes were obtained by Illumina NGS. Genome comparative analysis showed their quadripartite structure and their genetic diversity was assessed, including the identification of repeated regions (SSRs, large sequence repeats, among others). Barcode DNAs were developed in hypervariable regions for species molecular identification. Furthermore, the phylogenetic analysis revealed the evolutionary relationships of *Ficus* species, shedding light in the actual controversy among others.

## Results

### Features of the *Ficus* species chloroplast (CP) genome

**Fig. 1:**
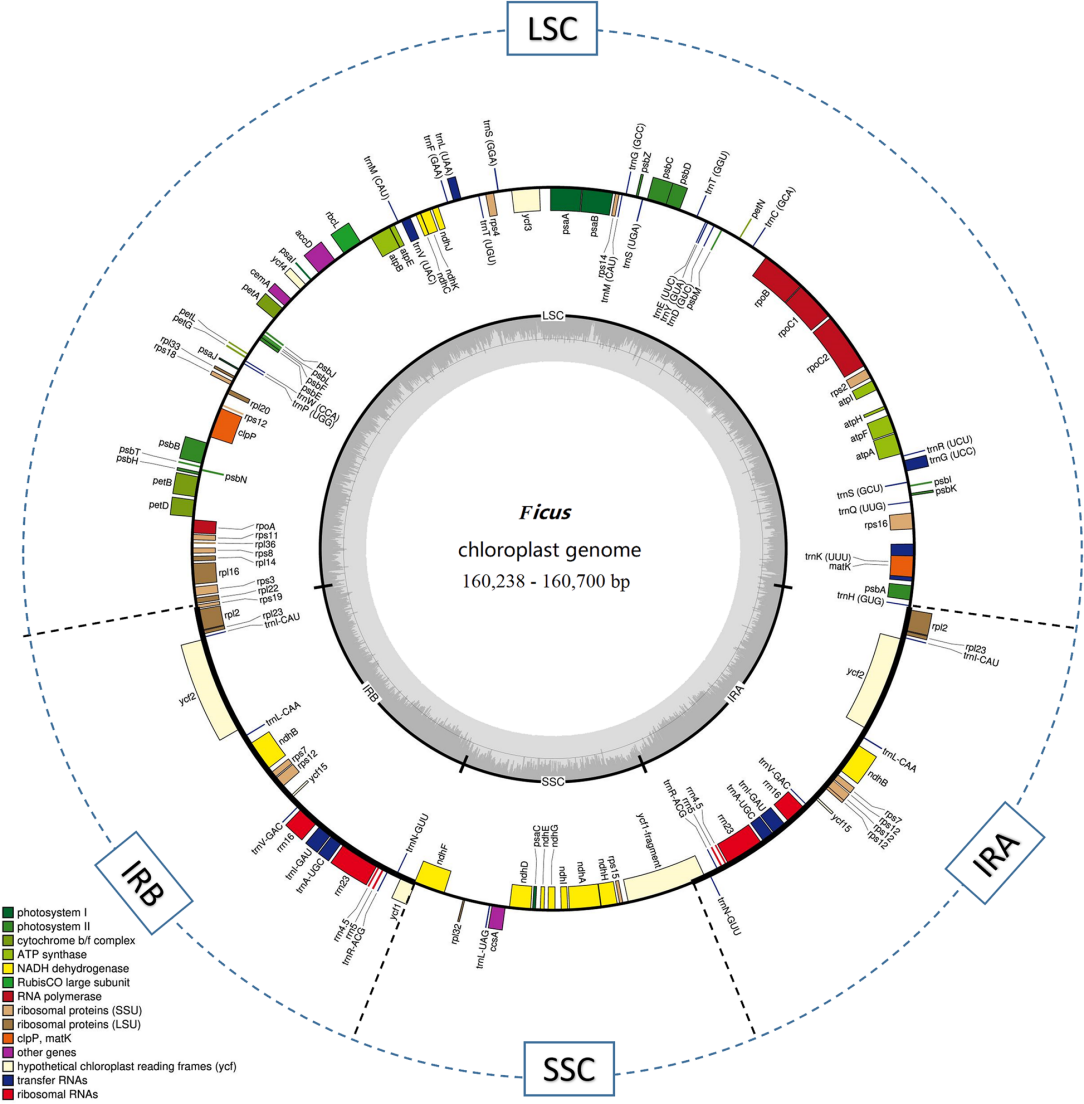
Genome map of the average *Ficus* CP genome obtained in this work. The inner circle represents the quadripartite structure, with two copies of the inverted repeat (IRA and IRB), an LSC, and an SSC region in black with GC content in dark grey and AT content in light grey. External circle represents gene content, with those inside the circle transcribed clockwise, while the ones located at the outer side are counter clockwise transcribed. Genes are coloured following functional groups according to the legend show on the left bottom

The studied *Ficus* CP genomes display a typical circular double-chain structure, with sizes ranging from 160,238 to 160,700 bp (Fig. 1, Table 1). The *Ficus* plastomes show the classic quadripartite architecture, with an LSC region (88,400–88,804 bp) and an SSC region (19,926–20,145 bp) separated by two inverted repeat (IR) regions (25,840–25,901 bp). All eleven CP genomes show similar total GC content (ranging from 35.88% to 36.02%), being significantly higher in the IR regions (Table 1).

**Table 1:** Summary features of the *Ficus* species CP genomes characterized

Species	Total cp genome size(bp)	LSC length (bp)	IR length (bp)	SSC length (bp)	Total GC content (%)	LSC GC content (%)	IR GC content (%)	SSC GC content (%)
*F. pumila*	160,279	88,400	25,889	20,101	35.98	33.64	42.65	29.05
*F. tikoua*	160,700	88,804	25,876	20,144	35.88	33.52	42.63	28.92
*F. hispida*	160,323	88,533	25,840	20,110	35.92	33.57	42.65	28.95
*F. virens*	160,501	88,593	25,885	20,138	35.90	33.54	42.62	28.95
*F. sarmentosa* var. *impressa*	160,447	88,645	25,864	20,074	36.02	33.68	42.68	29.14
*F. sarmentosa* var. *lacrymans*	160,374	88,524	25,893	20,064	35.95	33.62	42.65	28.95
*F. pandurata*	160,644	88,701	25,899	20,145	35.88	33.50	42.63	28.97
*F. tinctoria*	160,366	88,508	25,878	20,102	35.94	33.58	42.69	29.03
*F. formosana*	160,463	88,518	25,901	20,143	35.90	33.56	42.62	28.91
*F. microcarpa*	160,238	88,540	25,886	19,926	35.93	33.59	42.60	28.97
*F. simplicissima*	160,375	88,446	25,897	20,135	35.92	33.56	42.66	28.97

While only counting one copy of those duplicated genes in the IR region, we annotated a total of 114 unique genes, consisting of 30 tRNAs, 4 rRNAs, and 80 protein-coding genes in each of the *Ficus* plastomes characterized. Furthermore, the overall length of the CDS region ranged from 80,334 to 80,598 bp. And the content of GC in CDS regions is slightly higher than that of the whole, varying from 37.1 to 37.2% (Table S1). In detail, in all eleven CP genomes, we identified 16 duplicated genes in the IR region, among which there are seven tRNA genes, four rRNA genes, and five protein-coding genes. A total of 63 CDS and 22 tRNA genes are present in the LSC region, while 12 CDS and one tRNA gene exist in the SSC region (Table S2). Two pseudogenes (*ycf1* and *rps19*) are located in the boundary between IR-SSC and IR-LSC.

There were 18 genes harbouring introns, which can regulate gene expression and enhance the expression of exogenous genes at specific sites and specific times of the development of the plant [29, 30]. Among those, 12 are protein-coding genes and 6 are tRNA genes. Most genes [15] have only a single intron, whereas *ycf3* and *clpP* genes contain two introns. The *rps12* gene is so unique that it is composed of three complex exons, containing one 5’ exon and two 3’ exons. The 5’ exon is located in the LSC region, while the 3’ exons are distributed within the IR regions, which is consistent with close species such as *Ficus religiosa* [31], *Morus celtidifolia* [32], and *Broussonetia kazinoki* [33]*.* Two pseudogenes, *ycf1* and *rps19,* are located between the IRB/ SSC and IRA /LSC, respectively. On account of the reverse repeating property of the IR region, these two genes fail to be fully duplicated and lose the ability to encode a complete protein, which leads to the presence of two pseudogenes. In addition, the *trnK-UUU* gene, which embodies the *matK* gene, has the largest intron (2,583–2,601 bp), compared to other genes (Table S3).

### Identification of repeat elements

**Fig. 2:**
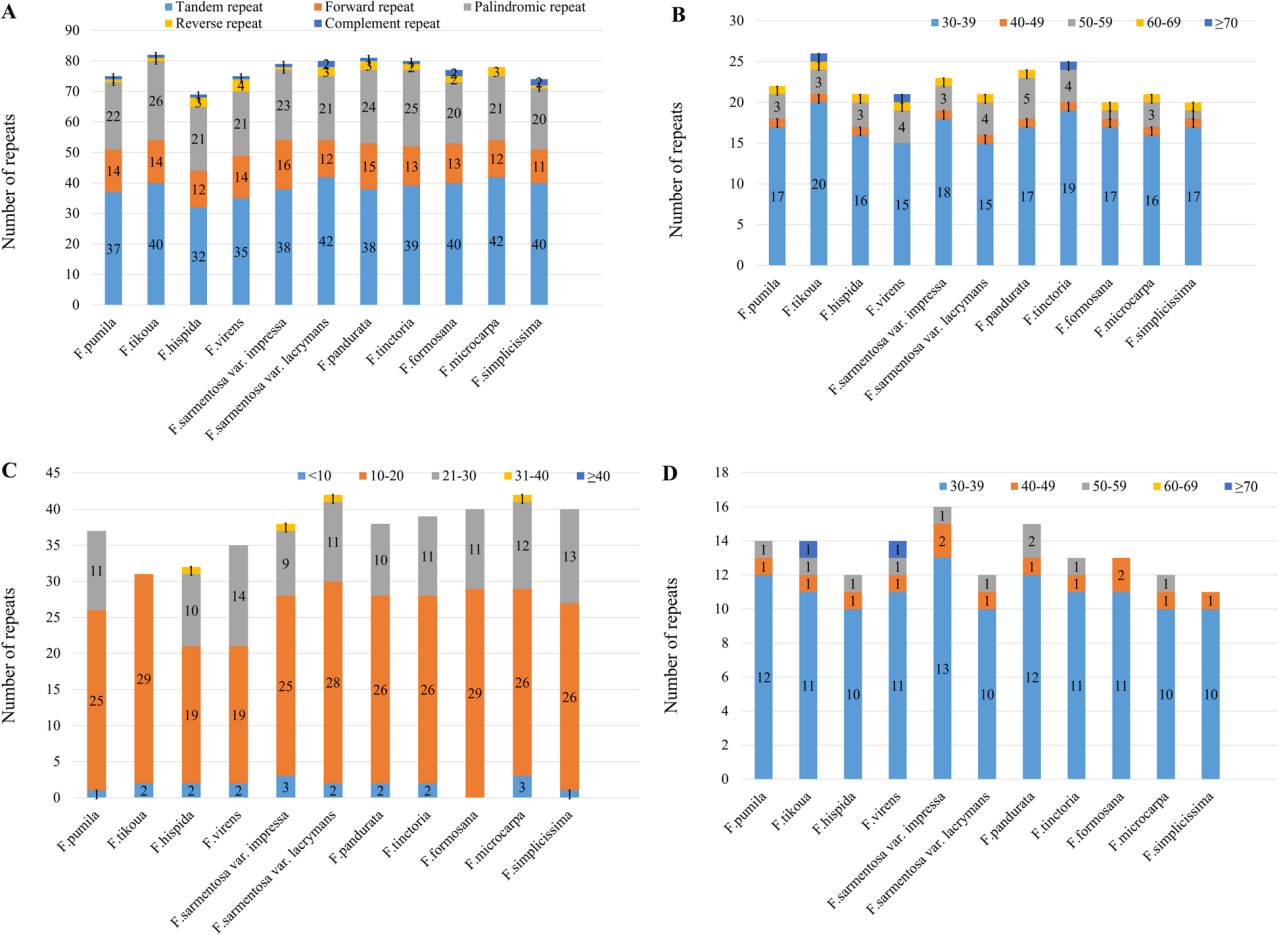
Repeat sequences analysis in eleven *Ficus* plastomes. A: Repeat types of eleven CP genomes. B: Tandem repeats in eleven CP genomes. C: Palindromic repeats in eleven CP genomes. D: Forward repeats in eleven CP genomes. Repeats with different lengths are indicated in different colours, the ordinate represents the number of repeats

A mass of repeated sequences is widely distributed in the intergenetic spacer and intron sequences of the *Ficus* CP genome, which have always been the focus of genome research [34, 35]. Long repeats with a length greater than 30 bp might have functions in promoting chloroplast genome rearrangement and increasing population genetic diversity [36]. For the purpose of getting a comprehensive understanding of the long repeats within the *Ficus* CP genome, we classified those repeated sequences into five categories, namely tandem, forward, palindromic, reverse, and complementary repeats. These results manifested that the number of repeated sequences in the eleven *Ficus* CP genomes ranges from 69 (*F. hispida*) to 82 (*F. tikoua*). Among them, the number of tandem repeats were found to be the most abundant (46.4%-54.1%), varied from 32 (*F. hispida*) to 42 (*F. sarmentosa* var. *lacrymans, F. microcarpa*), followed by palindromic repeats (26.0%-31.7%), ranging from 20 (*F. formosana, F. simplicissima*) to 26 (*F. tikoua*), and then by forward repeats (14.9%-20.3%), with the scope of 11 (*F. simplicissima*) to 16 (*F. sarmentosa* var. *impressa*) (Fig. 2A). Among the ten *Ficus* species, the length of tandem repeats is generally distributed between 10 and 20 bp, while the size of palindromic and forward repeats is concentrated between 30 and 39 bp (Fig. 2B-D).

**Fig. 3:**
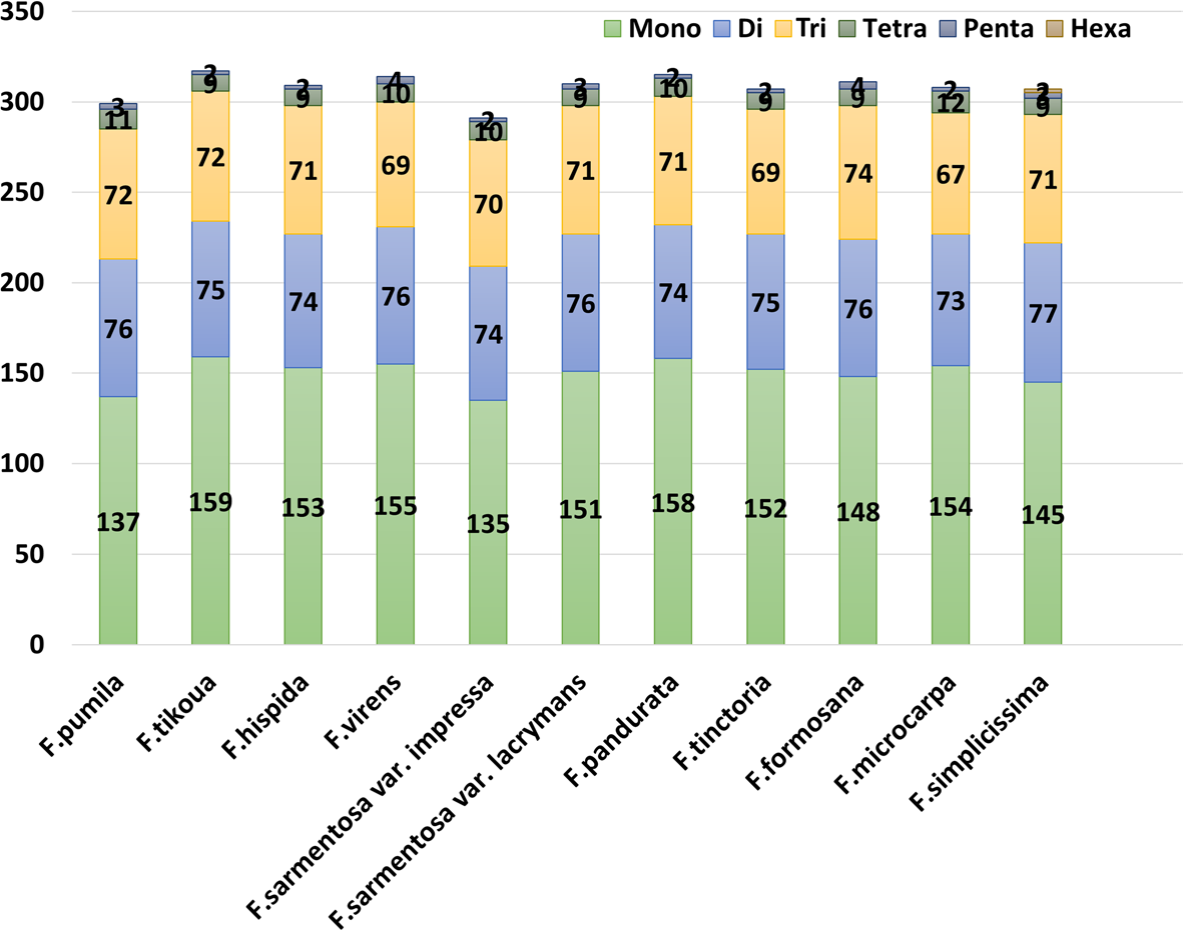
Analysis of number and type of SSRs in eleven *Ficus* plastomes. SSRs with different types are indicated in different colours

Simple sequence repeats (SSRs) are composed of small repeated sequences ranging from 1 to 6 bp [37], which are extensively distributed at different locations such as intergenic region, intron region, and even protein-coding region [38]. The CP genome possesses the nature of uniparental inheritance, leaving SSRs a high level of variation within the same species [39]. Thus, chloroplast SSRs are important sources for developing molecular markers, which are widely used in phylogenetic and population genetic analysis [40, 41]. Here, a total of 299–317 SSRs were identified in these *Ficus* plastomes (Fig. 3), with average percentages of mononucleotide, dinucleotide, trinucleotide, tetranucleotide SSRs being 48.59%, 24.39%, 24.94%, and 3.16%, respectively. It can be found that pentanucleotide SSRs are very rare in all sequenced genomes, and we were able to detect hexanucleotide SSRs only in the plastome of *Ficus simplicissima*.

### Codon usage and RNA editing sites

**Fig. 4:**
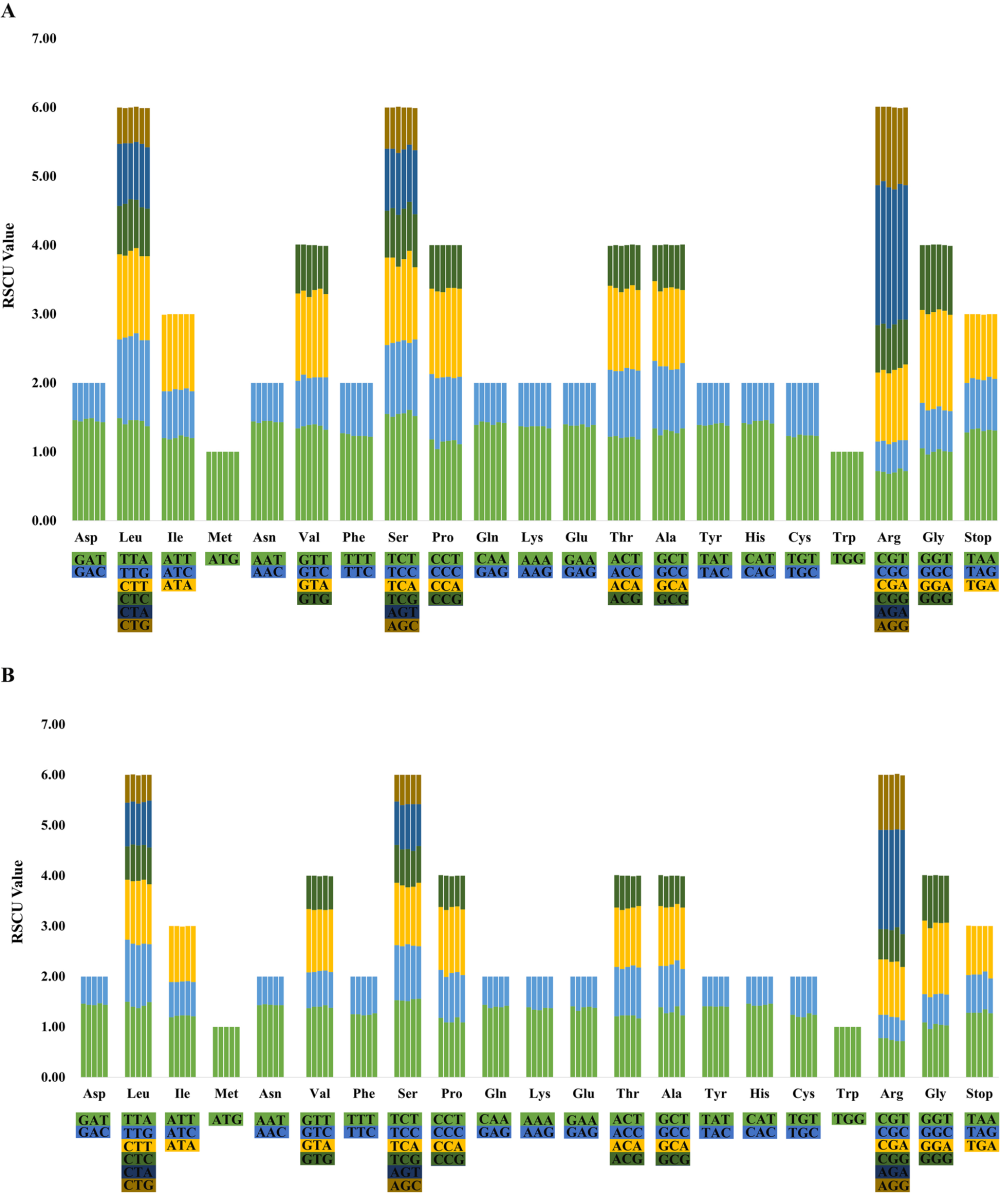
Codon content for the 20 amino acids and stop codons of CDS of the *Ficus* species CP genome. A: Codon content for CDS in the six *Ficus* CP genomes, each column in the bar graph represents a species. The corresponding species from left to right are *F. pumila*, *F. tikoua, F. hispida, F. virens, F. sarmentosa* var. *impressa,* and *F. pandurata*. B: Codon content for CDS in the rest five *Ficus* CP genomes, the corresponding species from left to right are *F. microcarpa, F. formosana, F. sarmentosa* var. *lacrymans, F. simplicissima,* and *F. tinctoria*

Codon usage patterns and nucleotide composition help to lay a theoretical foundation for genetic modifications of the CP genome [42, 43]. Here, amino acid frequency, codon usage number, and the relative synonymous codon usage (RSCU) in the eleven *Ficus* plastomes were analysed and summarized (Fig. 4, Table S4). A total of 64 RSCU were presented in the *Ficus* plastomes, and the number of codons varied from 53,412 to 53,566. Leucine and cysteine were the most and least universal amino acids, with UUU (encoding phenylalanine) and GCG (encoding alanine) as the most and least used codons in *Ficus,* respectively. Most of amino acid codons, except for methionine and tryptophan, had more than one synonymous codon, among which, leucine, serine, and arginine showed the maximum (six codon usage). Preferred codon is defined when its RSCU value was greater than 1.00. In the studied eleven *Ficus* plastomes, the number of preferred codon usage identified ranged from 28 to 32 (Fig. 4). Moreover, many of the preferred codons end with an A or T, whereas non-preferred codons ended with a C or G, supporting the reduced GC content in coding regions. This phenomenon is common in chloroplast genomes from other species [44, 45].

Previous studies have shown that the distribution of chloroplast RNA editing sites is uneven and more prone to protein-coding genes [46]. A total of 35 protein-coding genes were evaluated with the PREP program, to predict RNA editing sites in the *Ficus* plastomes. In sum, 59–65 RNA editing sites were identified (Table S5), in which amino acid conversion from S to L occurred the most frequently, while R-G occurred the least. Interestingly, it was found that all RNA editing sites appeared in the first position or second position of the corresponding codon, while no potential RNA editing sites were observed at the third position. The base conversion type is all from C to T, which is similar to those of other land plants [47, 48].

### IR contraction and expansion in the *Ficus* CP genome

**Fig. 5:**
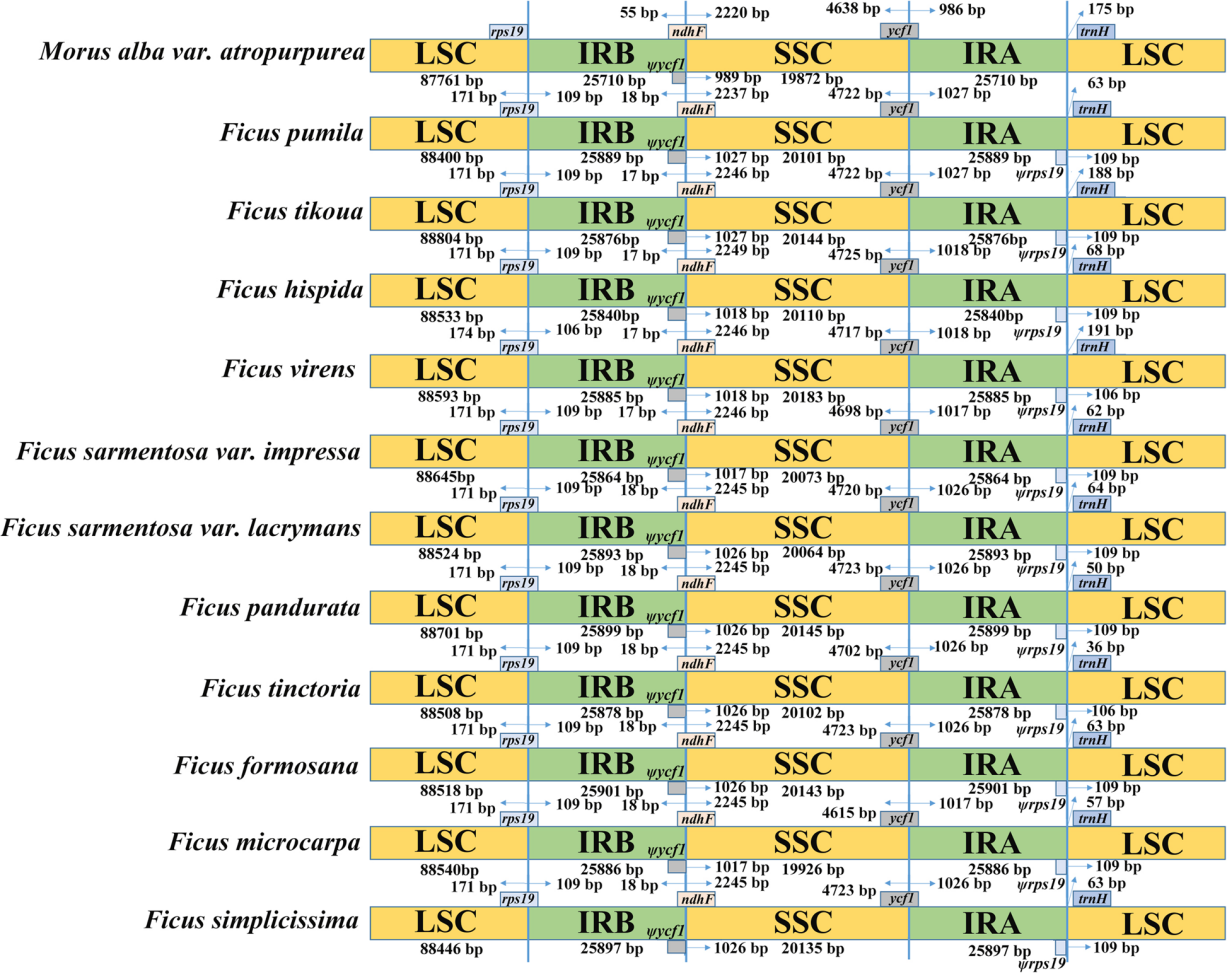
Comparison of the borders of LSC, SSC, and IR regions among twelve CP genomes. Corresponding species from top to bottom are *Morus alba* var. *atropurpurea, F. pumila, F. tikoua, F. hispida, F. virens,* and *F. sarmentosa* var. *impressa, F. sarmentosa* var. *lacrymans, F. microcarpa, F. pandurata, F. tinctoria, F.formosana, F. microcarpa,* and *F. simplicissima.* Ψ: pseudogenes

The typical quadripartite structure of the CP genome results in four boundary limits among IR, LSC, and SSC regions, namely IRB-LSC, IRB-SSC, SSC-IRA, and IRA-LSC [49, 50]. Although the inverted repeat regions (IRA and IRB) are the most conserved regions of the CP genome, shrinkage and expansion of the IR boundaries are hypothesized to help explain size differences between CP genomes beyond genus. The length of the IR region in the twelve CP genomes exhibited a modest expansion, ranging from 25,710 bp to 25,901 bp. In this work the IR-SSC and IR-LSC boundaries of *Ficus* species were compared to that of *Morus alba* var. *atropurpurea* (belonging to another genus within the Moraceae). Four affected protein coding genes that create some variable regions were found useful for species identification (Fig. 5).

In *M. albo* var. *atropurpurea*, the *rps19* gene is entirely located within the LSC region, while it expands to the IRB region in the studied eleven *Ficus* plastomes, altering the boundary LSC-IRB. This fact resulted in truncated *rps19* copies (*ψrps19*) at the junction IRA-LSC in *Ficus* species. Another gene crossing junction border is *ycf1* that crosses the IR-SSC borders within the twelve CP genomes, creating truncated *ψycf1* at the joint of IRB-SSC with a size variation from 986 to 1027 bp. It has been reported that the *ycf1* gene contributes to the analyses of the CP genome variation in higher plants. Another affected gene, *ndhF,* covers the IRB-SSC region exhibits high similarity in ten *Ficus* species. Whereas the *trnH* gene was found to be shifted from the IRA-LSC border in all twelve species, with the longest distance (118 bp) to the border observed in *F. tikoua* species (Fig. 5)*.*

### Comparative genomic analysis

Interspecific comparisons employing the online software mVISTA were performed to reveal the conservation and divergence among *Ficus* species, as previously done with other species [39, 51]. The eleven *Ficus* plastomes were compared to the *F. pumila* plastome as the reference (Fig. S1). We found that the two IR regions were less divergent than the LSC and the SSC regions, which also occurred in almost higher plants [52]. Moreover, the non-coding region exhibited more nucleotide divergence than the coding regions. In the coding region, most genes were relatively conservative except *matK, rps16, rpoC2, psbD, ndhD,* and *ycf1.* These divergence hotspot regions identified in the eleven plastome sequences provided vast information for the development of molecular markers for phylogenetic analyses and for *Ficus* plant species identification.

### Divergence hotspot region

**Fig. 6:**
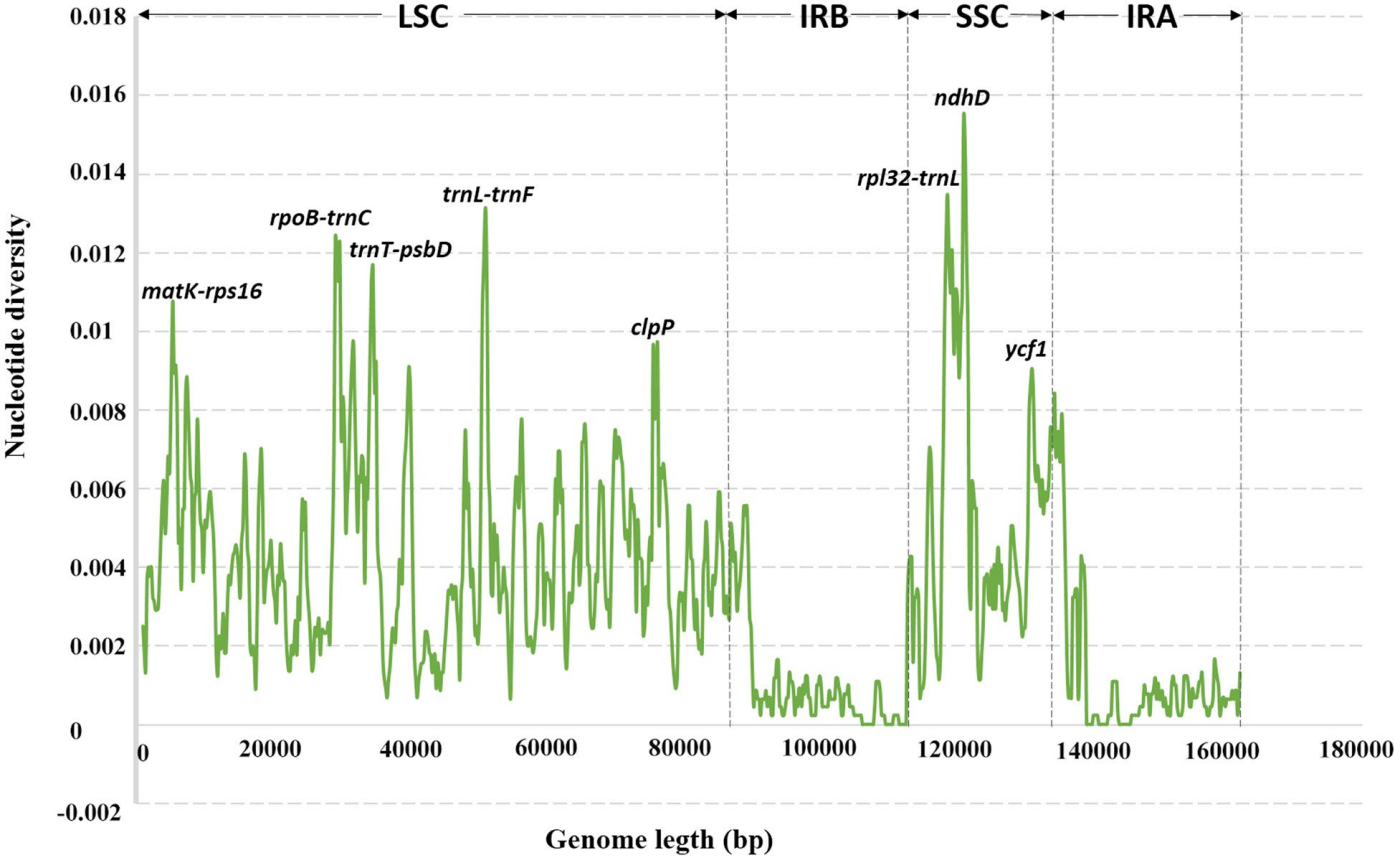
Comparative analysis of the nucleotide variability by Pi values of the eleven CP genomes presented in a sliding window (window length: 800 bp; step size: 200 bp). X-axis: position of the midpoint of a window; Y-axis nucleotide diversity in each window

Highly variable sequences can be utilized to determine the phylogenetic relationship between species and genera [53, 54]. Nucleotide diversity (Pi) values were calculated within 800-bp windows (Fig. 6) to identify sequence divergence hotspots. The result showed that the Pi value of the whole Ficus CP genome varied from 0 to 0.01543, which represents the nucleotide diversity. Eight highly variable regions (Pi > 0.009) were detected: *matK-rps16, rpoB-trnC, trnT-psbD, trnL-trnF, rpl32-trnL, clpP, ndhD* and *ycf1*. Among these, five regions (*matK-rps16, rpoB-trnC, trnT-psbD, trnL-trnF,* and *clpP*) are located in the LSC region, and the remaining three are in the SSC region (Fig. 6). This is consistent with preceding results that the IR region is generally more conserved than the LSC and the SSC regions [34, 55].

### Phylogenetic analysis

**Fig. 7:**
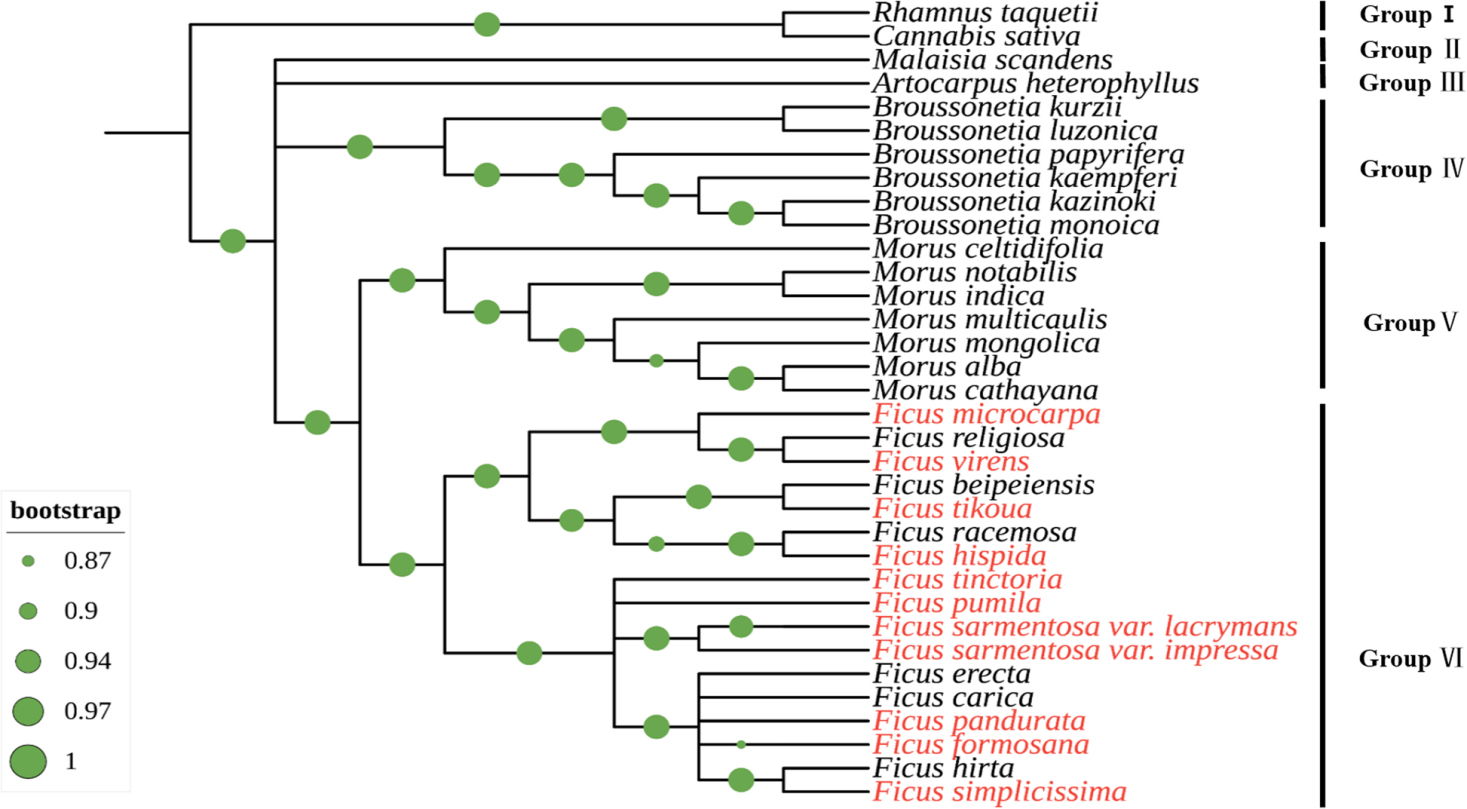
Phylogenetic relationships among 34 plant species based on CP genome. Phylogenetic inference was performed with concatenated protein coding genes sequences from all species shown using ML method, with branch support shown as Bootstrap values with green circles

Phylogenetic analysis is often used to infer or evaluate evolutionary relationships [28, 56]. To examine the phylogenetic positions of the ten *Ficus* species and their relationships within Moraceae, ML phylogenetically analyses was performed using concatenated protein coding genes sequences from 32 CP genomes belonging to 5 genera of Moraceae and two CP genomes beyond the Moraceae family. As illustrated in Fig. 7, the phylogenetic tree has divided all species into six groups (I to VI), with almost all nodes supported with 100% bootstrap values (BP). Group I contained two species (*Cannabis sativa* and *Rhamnus taquetii*), which were set as outgroups. Those Moraceae species shaped into four paraphyletic groups. Group II and Group III consisted of *Malaisia scandens* from the genus *Malaisia*, and *Artocarpus heterophyllus* from the genus *Artocarpus*, respectively. Group IV contained six species belonging to the genus *Broussonetia* and Group V correspond to seven species from the genus *Morus*. Group VI was the most complex, and the real target of this study, which was comprised of 17 species from the genus *Ficus* and was further divided into three subgroups, each belonging to a different subgenus. The first subgroup contained *F. microcarpa* and *F. virens*, being clustered with *F. religiosa* which belongs to the subgenus *Urostigma*. The second subgroup contained *F. tikoua* and *F. hispidia*, being clustered with subgenus *Sycomorus* species (*F. racemosa* and *F. beipeiensis*). Whereas the third subgroup clustered 9 species belonging to *Ficus* subgenus, of which 6 species (7 sequences) were obtained in this work (Fig. 7). The Ficus clade was sistered to the Morus clade, whose common ancesteor derives from Goup II to IV, indicating a close relationship between the Ficus and Morus genera.

## Discussion

### *Ficus* plastomes characterization and use for species identification

Eleven *Ficu*s CP genomes were obtained and analysed in this study. The comparative analysis revealed highly conserved structures and genes. The plastome sizes showed slight differences, which suggested that the CP genome length in *Ficus* is highly conserved.

Repeat sequences, which are dispersed in CP genomes at high frequency, play a vital role in genome organization and evolution. In this work, we found resembling repeat types with similar distributions among ten *Ficus* species. SSRs, displaying a high level of polymorphism, are common in the CP genome as microsatellite repeats [38]. These sequences were used as a genetic marker in previous investigations [57]. The SSRs in the *Ficus* CP genomes were found to be particularly rich in AT, which corresponded with previous studies where proportions of polyadenine (polyA) and polythymine (polyT) were higher than polycytosine (polyC) or polyguanine (polyG) within chloroplast SSRs in many plant species [58].

RNA editing is a very common phenomenon that exists in plant CP genomes. The main functions of RNA editing include modifying mutations, correcting and regulating translation [59]. Interestingly, among the 35 protein-coding genes used to predict RNA editing sites, *ndhB* and *ndhD* have the most editing sites, and both encode subunits of the chloroplast NADH dehydrogenase complex, which is involved in electron transfer during photosynthesis [60].

The expansion and contraction of IR and SC (including LSC and SSC) boundaries are thought to be the main cause of CP genome size changes, although CP genomes in land angiosperms are highly conserved [61]. After comparing CP genomes among the ten *Ficus* species in our study, we found that the boundary region between the SC and two IR regions was relatively conserved, with gene distribution and specific location exhibiting high consistency. Compared with the other *Morus* species from the same family, the IR region of *Ficus* species showed expansion, mainly because the *rps19* gene located at the LSC-IR boundary, expanded to the IR region by 109 bp. This indicates that the contraction and expansion of the IR regions are more common among different genera.

DNA barcoding is a method for rapid and accurate identification of species using a short and accurate DNA fragment. The concept of DNA barcoding was first proposed in 2003 by Hebert et al. [62]. Since then, an increasing number of researchers have focused on the selection of one or a few standard markers as DNA barcode(s). The earliest proposed DNA barcoding technology can identify species through *ITS2*, *matK*, *psbA-trnH*, *rbcL* and other DNA sequences [63]. However, it was found that these classical DNA barcodes were not suitable for the identification of the *Ficus* species of this study, due to the low nucleotide diversity in those ‘universal’ barcode fragments. Hence, finding suitable DNA markers for proper identification of these species was crucial. Here, according to nucleotide diversity analysis shown in Fig. 6, eight regions arose as putative barcoding regions, including five intergenic regions (*matK-rps16*, *rpoB-trnC*, *trnT-psbD*, *trnL-trnF,* and *rpl32-trnL*) and three genic regions (*clpP*, *ndhD,* and *ycf1*). Among these regions or genes, the *ycf1* gene, as the second-largest gene in the chloroplast genome, is crucial for plant viability. Dong et al. [64] have proposed that the *ycf1* is the most variable site in the chloroplast genome, showing greater variability than existing chloroplast candidate barcodes such as *rbcL*, *matK*, and *trnH-psb*A, and thus may have potential applications as land plant DNA barcodes. Another two genic markers *clpP* [65] and *ndhD* [66] have also been reported as a region of high variation for plant molecular identification.

Five intergenic spacer regions including *matK-rps16*, *rpoB-trnC*, *trnT-psbD*, *trnL-trnF* and *rpl32-trnL*, located within the SSC, are highly variable regions in the *Ficus* chloroplast genome, which have also been proposed as potential DNA barcodes in other species. Among them, *matK-rps16* was demonstrated well utilization as DNA barcodes for *Triticum* plant [67] and *rpoB-trnC* was identified to be an effective marker for three *Synstylae* species [68]. Cheng et al. [69] suggested that *trnT-psbD* and *rpl32-trnL* potentially be used as molecular genetic markers for population genetics and phylogenetic studies of *E. mollis.* And *trnL-trnF* has a long history of use in plant phylogenetic studies [70], whereas this spacer often contains large A/T-rich regions that may lead to a low sequence quality [71]. Generally, although several candidate barcoding regions were identified, further research is still necessary to determine whether these highly divergent markers could be used in the identification and phylogenetic analyses of *Ficus* species.

### *Ficus* phylogenetic relationships with other members of Moraceae family

The Moraceae family consists of approximately 40 genera with 1100 species, most of which are distributed in tropical and subtropical regions [72]. It mainly includes genus *Ficus, Malaisia, Artocarpus, Broussonetia, Morus* among others. At present, little research has been reported on the phylogeny of Moraceae, especially focusing on *Ficus* species. *Ficus*, regarded as a model system for understanding co-evolution dating back more than 75 million years, has not been able to confidently resolve phylogenomic relationships due to the lack of well-supported phylogenetic hypothesis, lack of species involved in the study or reduced dataset [73, 74]. Previously, Herre et al. (1996) performed the molecular phylogenetic studies of 15 *Ficus* species based on *trnL-F* and *rbcL* chloroplast markers [75]. Then, Renoult et al. (2009) revealed the potential of five non-coding chloroplast markers to address deep phylogenetic relations in *Ficus,* accounting for 38 species of African *Ficus* from the *Urostigma* section of *Galoglychia* subgenus [76]. Appearing significant conflicts when *Ficus* plastid phylogeny was compared with *Ficus* phylogeny based on ribosomal ITS and ETS [77]. These studies failed to represent what we currently know about the phylogenetic diversity within *Ficus*, and only sampled a maximum of 3,604 bp of plastid DNA [75, 76, 78]. More recently, Bruun-Lund et al. (2016) have examined the chloroplast genomes of 59 *Ficus* species and revealed that the phylogenies built from these genetic data provided both additional support to the current understanding of the evolutionary relationship of major species groups and discordance with information inferred from nuclear data [31]. In this study, eleven new CP genomes from ten *Ficus* species were added to solve this controversy, finding that *Morus* and *Ficus* are closely related compared with other genera. The eleven new CP genomes clustered into the same clade, with other reported *Ficus* species and can be distinguished from other genera of the *Moraceae* family (see Fig. 7). Furthermore, our study allowed unveil clustering of species within species from subgenus *Urostigma*, subgenus *Sycomorus*, and subgenus *Ficus*, all with high bootstrap values. These results support Berg classification system [2], in which the subgenus *Ficus* was further divided into the subgenus *Sycomorus*, and also support species differentiation based on molecular data. For example, *F. tikou*a, belonging to the *Ficus* subgenus based on morphology, helped to further differentiate a monophyletic group separating members of *Ficus* subgenus. Overall, these results are helpful to further understand the phylogenetic status and resolve relationships deep within *Ficus*.

## Conclusions

In conclusion, in this work we determined the complete plastome sequence of ten *Ficus* species by NGS. Comparative genomics indicate that these plastomes showed the typical quadripartite structure being relatively conserved, with eight mutation hotspot regions being presented as potential molecular markers for subsequent *Ficus* species identification. The phylogenomic analysis performed clarified the taxonomy of the species, showing the relatedness between *Ficus* and *Morus* genera, and the split of Ficus genus into three subgenera (*Ficus, Sycomorus* and *Urostigma)*. All together, these results enrich the data on the CP genome of the genus *Ficus* and provide additional information for future species identification and phylogenetic reconstruction of the *Ficus* species.

## Materials and methods

### Plant material, DNA extraction, and sequencing

Ten species (one of them contains two varieties), namely *F. pumila, F. tikoua, F. hispida, F. virens, F. sarmentosa* var. *impressa, F. sarmentosa* var. *lacrymans, F. pandurata, F. tinctoria, F. formosana, F. microcarpa,* and *F. simplicissima* were field-collected from the Medicinal Botanical Garden of Guangzhou University of Chinese Medicine with Longitude 113°24’ and Latitude 23°03’ (Guangzhou, Guangdong, China,). The formal identification of the plant material was undertaken by Dr. Jiaxia Su (Guangzhou University of Chinese Medicine). Permission was not necessary for collecting these species, which have not been included in the list of national key protected plants. Fresh green leaves cleaned with 75% ethanol from those collected *Ficus* plants were sampled. Then those leaves were dried and stored at -80 °C till DNA extraction. Total genomic DNA was extracted from 100 mg of cleaned leaves using a DNeasy Plant Mini Kit (Qiagen, German). Then, genomic DNA was examined for purity and integrity by ultraviolet spectrophotometry and gel electrophoresis (1 × TAE agarose gel), respectively.

High quality DNA was sheared to 500 bp using an ultrasonic DNA fragmentation apparatus (Covaris). Libraries were constructed with NEB Next Ultra DNA Library Prep Kit (New England Biolabs, E7370L) following the manufacturer’s protocol by the Sangon biotech High-Throughput DNA Sequencing Center. Libraries were amplified with NEB Next Q5 Hot Start HiFi PCR Master Mix kit, quantified on a Qubit 4.0 fluorometer (Thermo) and quality checked on an Agilent Technologies 2100 Bioanalyzer, prior paired-end 150 × sequencing in Illumina Hiseq 4000 sequencing platform at the Sangon biotech Sequencing Center.

### Chloroplast genome assembly and annotation

After Illumina sequencing (paired-end, 150 ×), approximate 15 Gb of raw data for each sample was generated, and these raw reads were QC filtered and trimmed using the Trimmomatic (v0.39, Max Planck Institute of Molecular Plant Physiology, Potsdam, Germany) software [79] with following parameters: LEADING = 20, TRAILING = 20, SLIDINGWINDOW = 4:15, MINLEN = 36, and AVGQUAL = 20. A more detailed information related to quality control of the Illumina sequencing of the chloroplast genome of *Ficus* species is shown in Table S6. Taking the complete sequence of *Ficus religiosa* chloroplast genome (downloaded from NCBI with GenBank accession number: NC_033979) as the reference, CP-like reads were extracted from those clean reads by mapping with the bwa software (v0.7.17) [80]. Next, these CP-like reads were assembled using the SPAdes (v3.13.1) program [81], obtaining several contigs. Contigs were mapped against the *F. religiosa* reference genome with mummerplot (v3.5) to form a complete chloroplast genome sequence with their overlapping sequences. BLASTn (2.8.1) was conducted for self-alignment to locate the precise position of the quadripartite structure. Four regions between the IR regions and the LSC/SSC region were amplified and sequenced using specific primers (Table S7) in order to verify each CP assembly. A preliminary *Ficus* plastomes gene annotation was performed with the GeSeq online tool (https://chlorobox.mpimp-golm.mpg.de/geseq.html) with default parameters [82]. The annotation results were further examined and revised manually, according to reference genomes with the CLC Sequence Viewer. A detailed CP genome map for each *Ficus* species was drawn using the Organellar Genome DRAW (OGDRAW) v1.2 (Max Planck Institute of Molecular Plant Physiology, Potsdam, Germany) [83].

### SSRs and repeat sequence analysis

Repeat sequences (including forward, reverse, complementary, palindromic) were identified by running the REPuter tools (https://bibiserv2.cebitec.uni-Bielefeld.de/reputer) [84] with a Hamming distance set at 3 and a minimum repeat size of 30 bp. Tandem repeats were analysed by the Tandem Repeats Finder (http://tandem.bu.edu/trf/trf.html), with alignment parameters set to 2, 7, and 7 for matches, mismatches, and indels. Whereas MISA was used to detect simple sequence repeats [85].

### Genome structure and genome comparison

Molecular Evolutionary Genetics Analysis software MEGA v. 11 [86] (https://www.megasoftware.net/) was used to analyse codon usage distribution, GC content and phylogenomic inference as described below. Thirty-five protein-coding genes of the chloroplast genome of those eleven *Ficus* plastomes were used to predict potential RNA editing sites using the online program Predictive RNA Editor for Plants (PREP) suite (Mower 2009), with a cutoff value of 0.8. The mVISTA program (http://genome.lbl.gov/vista/index.shtml) in the Shuffle-LAGAN mode was used to align the obtained *Ficus* CP genomes with one reported CP genome (*Morus atropurpurea*) within the Moraceae family, whose sequence was downloaded from NCBI (GenBank accession number: KU355276) [87].

### Sequence divergence and phylogenetic analysis

MAFFT (v7.419) was employed to align the CP genome sequence of ten *Ficus* species and then adjusted manually by Se-Al 2.024 [88]. DnaSP v5.10 software [89] was used to identify rapidly evolving molecular markers that can be applied to further phylogenetic studies, with a sliding window analysis with the step size and window length set as 200 and 800 bp.

To illustrate the phylogenetic positions and evolutionary relationships of *Ficus* species within the Moraceae family, the complete CP genomes of 23 species (21 from five different genera within the Moraceae, with *Rhamnus taquetii* and *Cannabis carmagnole*, that were set as out-group) were downloaded from the GenBank of NCBI (Table S8). Maximum-likelihood (ML) phylogenetic inference analysis was performed on a nucleotide alignment of 80 protein-coding genes using MEGA v.11. In detail, an ML tree inference was conducted using the general time-reversible model with a gamma distribution of substitution rate among sites (GTR + G), which was selected according to a previous model screening analysis (Model test as implemented in MEGA v11). To optimize the ML method, TBR branch switching (a fast and efficient branch switching operation), was adopted to improve the initial evolutionary tree, applying also 1,000 replicates. Bootstrap analysis to determine the support of each branch. Nucleotide and phylogeny inference models were selected after model testing in MEGA v.11.

## Supplementary Information

**Additional file 1: Figure S1.** Sequence identity plot comparison of the eleven CP genome of the ten *Ficus* species using mVISTA.

**Additional file 2: Table S1.** Gene number and CDS nucleotide composition of the CP genomes in eleven *Ficus* species.

**Additional file 3: Table S2. **Gene contents in the *Ficus* species chloroplast genome.

**Additional file 4: Table S3.** Gene with introns in the *Ficus* species chloroplast genome and the lengths of the introns and exons.

**Additional file 5: Table S4.** Codon usage of the eleven *Ficus* chloroplast genomes.

**Additional file 6: Table S5.** Predicted RNA editing sites in eleven *Ficus* chloroplast genomes by the PREP program.

**Additional file 7: Table S6.** Quality control of the Illumina sequencing of chloroplast genome of *Ficus* species.

**Additional file 8: Table S7.** Universal primers for amplifying four regions between the IR regions and the LSC/SSC region.

**Additional file 9: Table S8.** The 23 studied species and the corresponding chloroplast whole genome GenBank accession number.

## Data Availability

The sequencing datasets generated during the current study are available at China National GeneBank with project number as CNP0001337 (https://db.cngb.org/search/project/CNP0001337/). The accession numbers of eleven species are CNS0285141 (*F. pumila*), CNS0285142 (*F. tikoua*), CNS0285143 (*F. hispida*), CNS0285144 (*F. virens*), CNS0285145 (*F. sarmentosa* var. *impressa*), CNS0285146 (*F. sarmentosa* var. *lacrymans*), CNS0285147 (*F. pandurata*), CNS0285148 (*F. tinctoria*), CNS0285149 (*F. formosana*), CNS0285150 (*F. microcarpa*) and CNS0285151 (*F. simplicissima*).

## Abbreviations

CP: Chloroplast
SSC: Small single copy
LSC: Large single copy
IR: Inverted repeat
SSR: Simple sequence repeat
SNPs: Single-nucleotide polymorphisms
CDS: Coding sequences
RSCU: Relative synonymous codon usage
BP: Bootstrap values
polyA: Polyadenine
polyT: Polythymine
polyC: Polycytosine
polyG: Polyguanine
