# Supplementary material for: Comprehensive analysis of complete chloroplast genome and phylogenetic aspects of ten Ficus species
Source: BMC Plant Biol. 2022 May 23;22:253. doi: 10.1186/s12870-022-03643-4 (PMC9125854; doi:10.1186/s12870-022-03643-4)
Supplement: Supplementary file 1 — Additional file 1: Figure S1. Sequence identity plot comparison of the eleven CP genome of the ten Ficus species using mVISTA. [file 12870_2022_3643_MOESM1_ESM.doc]

**
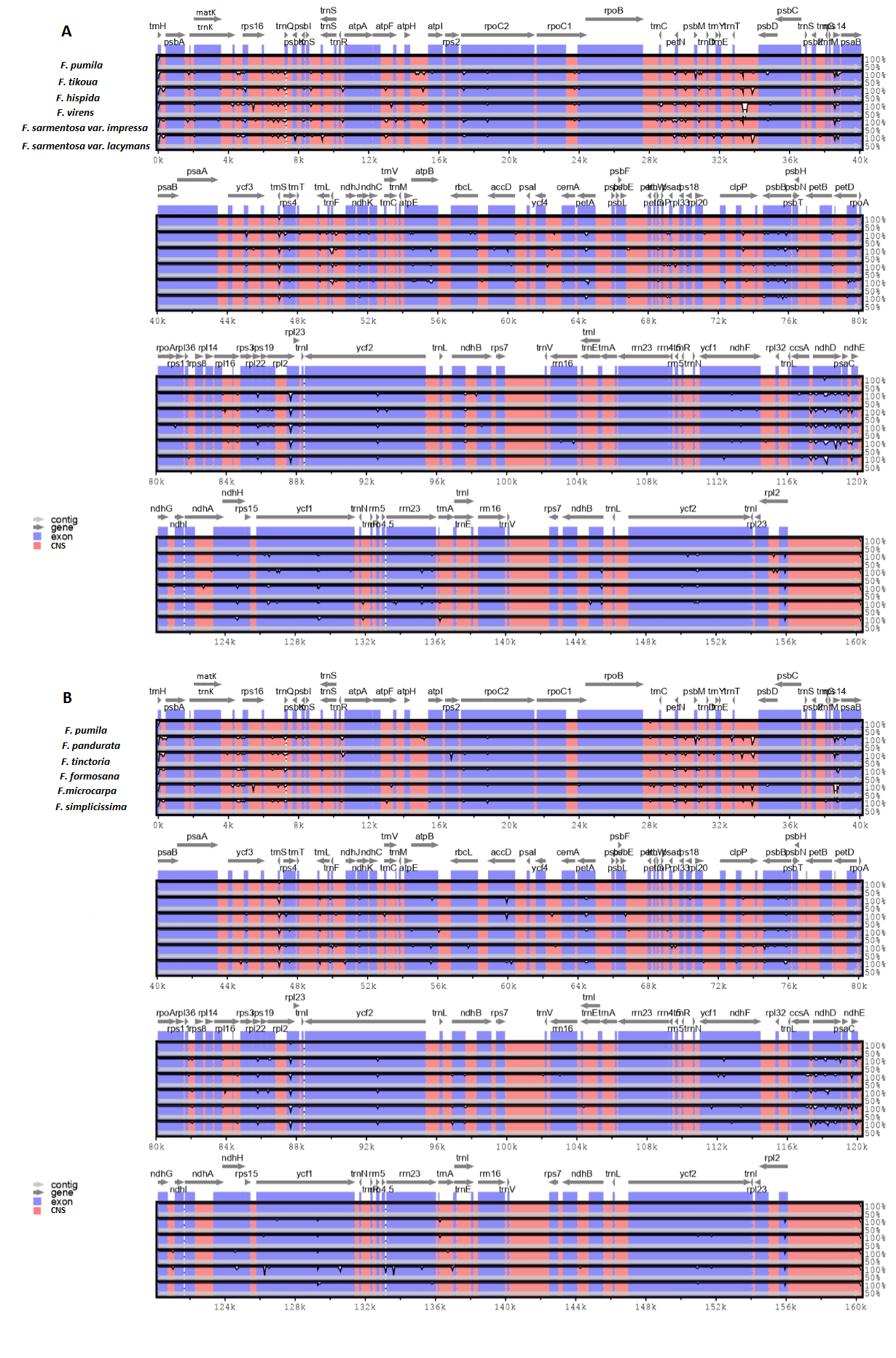
**

**Figure S1.** Sequence identity plot comparison of the eleven CP genome of the ten *Ficus* species using mVISTA. A: CP genome comparison of six *Ficus* species including *F. pumila*, *F. tikoua, F. hispida, F. virens, F. sarmentosa var. impressa and* *F. sarmentosa var. lacrymans.* B: Comparison of six *Ficus* CP genomes, the corresponding species from top to bottom are *F. pumila, F. pandurata, F. tinctoria, F. formosana, F. microcarpa* and *F. simplicissima*. Gray arrows and thick black lines above the alignment indicate genes with their orientation and the position of the IRs, respectively. A cut-off of 70% identity was used for the plots, and the Y-scale represents the percentage identity ranging from 50 to 100%.
