# Supplementary material for: Comprehensive analysis of complete chloroplast genome and phylogenetic aspects of ten Ficus species
Source: BMC Plant Biol. 2022 May 23;22:253. doi: 10.1186/s12870-022-03643-4 (PMC9125854; doi:10.1186/s12870-022-03643-4)
Supplement: Supplementary file 2 — Additional file 2: Table S1. Gene number and CDS nucleotide composition of the CP genomes in eleven Ficus species. [file 12870_2022_3643_MOESM2_ESM.doc]

**Table S1**. Gene number and CDS nucleotide composition of the CP genomes in eleven *Ficus* species

| **Species** | **Number of** | | | | **CDS length(bp)** | **ATCG contents in CDS (%)** | | | |
| --- | --- | --- | --- | --- | --- | --- | --- | --- | --- |
|  | unigenes genes | CDS | tRNAs | rRNAs |  | T | C | A | G |
| ***F.pumila*** | 114 | 80 | 30 | 4 | 80382 | 31.84 | 17.44 | 30.99 | 19.73 |
| ***F.tikoua*** | 114 | 80 | 30 | 4 | 80355 | 31.86 | 17.42 | 31.00 | 19.72 |
| ***F.hispida*** | 114 | 80 | 30 | 4 | 80334 | 31.87 | 17.41 | 31.00 | 19.71 |
| ***F.virens*** | 114 | 80 | 30 | 4 | 80426 | 31.86 | 17.42 | 31.02 | 19.70 |
| ***F.sarmentosa var. impressa*** | 114 | 80 | 30 | 4 | 80598 | 31.86 | 17.47 | 30.94 | 19.73 |
| ***F.sarmentosa var. lacrymans*** | 114 | 80 | 30 | 4 | 80445 | 32.59 | 17.49 | 30.24 | 19.68 |
| ***F.pandurata*** | 114 | 80 | 30 | 4 | 80397 | 31.85 | 17.43 | 31.00 | 19.71 |
| ***F.tinctoria*** | 114 | 80 | 30 | 4 | 80536 | 32.61 | 17.48 | 30.27 | 19.65 |
| ***F.formosana*** | 114 | 80 | 30 | 4 | 80550 | 32.59 | 17.47 | 30.28 | 19.66 |
| ***F.microcarpa*** | 114 | 80 | 30 | 4 | 80462 | 32.62 | 17.47 | 30.28 | 19.62 |
| ***F.simplicissima*** | 114 | 80 | 30 | 4 | 80446 | 32.58 | 17.50 | 30.26 | 19.66 |
