# Supplementary material for: Comprehensive analysis of complete chloroplast genome and phylogenetic aspects of ten Ficus species
Source: BMC Plant Biol. 2022 May 23;22:253. doi: 10.1186/s12870-022-03643-4 (PMC9125854; doi:10.1186/s12870-022-03643-4)
Supplement: Supplementary file 3 — Additional file 3: Table S2. Gene contents in the Ficus species chloroplast genome. [file 12870_2022_3643_MOESM3_ESM.doc]

| Gene Classification | Gene Names | Number |
| --- | --- | --- |
| Photosystem I | psaA,psaB,psaC,psaI,psaJ | 5 |
| Photosystem II | psbA,psbB,psbC,psbD,psbE,psbF,psbH,psbJ,psbK,psbL,psbM,psbN,psbT,psbZ | 15 |
| Cytochrome b/f complex | petA,petB,petD,petG,petL,petN | 6 |
| ATP synthase | atpA,atpB,atpE,atpF,atpH,atpI | 6 |
| NADH dehydrogenase | ndhA,ndhB(X2),ndhC,ndhD,ndhE,ndhF,ndhG,ndhH,ndhI,ndhJ,ndhK | 12（1） |
| RuBisCO large subunit | rbcL | 1 |
| RNA polymerase | rpoA,rpoB,rpoC1,rpoC2 | 4 |
| Ribosomal proteins(SSC) | rps2,rps3,rps4,rps7(X2),rps8,rps11,rps12(X2),rps14,rps15,rps16,rps18,rps19 | 14（2） |
| Ribosomal proteins(LSC) | rpl2(X2),rpl14,rpl16,rpl20,rpl22,rpl23(X2),rpl32,rpl33,rpl36 | 11 |
| Ribosomal RNAs | rrn4.5(X2),rrn5(X2),rrn16(X2),rrn23(X2) | 8（4） |
| Protein of unknow function | ycf1,ycf2（X2),ycf3,ycf4,ycf15(X2) | 7（2) |
| Transfer RNAs | 37 tRNAs | 37(7) |
| Other genes | accD,ccsA,cemA,clpP,matK | 4 |
| Total |  | 130 |

**Table S2.** Gene contents in the *Ficus species* chloroplast genome
