# Supplementary material for: Comprehensive analysis of complete chloroplast genome and phylogenetic aspects of ten Ficus species
Source: BMC Plant Biol. 2022 May 23;22:253. doi: 10.1186/s12870-022-03643-4 (PMC9125854; doi:10.1186/s12870-022-03643-4)
Supplement: Supplementary file 4 — Additional file 4: Table S3. Gene with introns in the Ficus species chloroplast genome and the lengths of the introns and exons. [file 12870_2022_3643_MOESM4_ESM.doc]

**Table S3** Gene with introns in the *Ficus* species chloroplast genome and the lengths of the introns and exons

| Species | Gene | Location | Exon I(bp) | Intron I(bp) | Exon II(bp) | Intron II(bp) | Exon III(bp) |
| --- | --- | --- | --- | --- | --- | --- | --- |
|  | atpF | LSC | 121 | 791 | 410 |  |  |
|  | clpP | LSC | 71 | 909 | 292 | 697 | 228 |
|  | ndhA | SSC | 552 | 1169 | 540 |  |  |
|  | ndhB | IR | 777 | 676 | 756 |  |  |
|  | petB | LSC | 6 | 802 | 642 |  |  |
|  | petD | LSC | 9 | 734 | 474 |  |  |
|  | rpl16 | LSC | 10 | 1078 | 398 |  |  |
| *Ficus* | rpl2 | IR | 390 | 685 | 435 |  |  |
| *pumila* | rpoC1 | LSC | 435 | 803 | 1626 |  |  |
|  | rps12 | LSC | 114 |  | 232 | 536 | 26 |
|  | rps16 | LSC | 42 | 891 | 228 |  |  |
|  | trnA-UGC | IR | 38 | 802 | 35 |  |  |
|  | trnG-UCC | LSC | 23 | 721 | 48 |  |  |
|  | trnI-GAU | IR | 37 | 945 | 35 |  |  |
|  | trnK-UUU | LSC | 35 | 2588 | 37 |  |  |
|  | trnL-UAA | LSC | 37 | 505 | 50 |  |  |
|  | trnV-UAC | LSC | 35 | 618 | 39 |  |  |
|  | ycf3 | LSC | 126 | 764 | 228 | 751 | 153 |
|  | atpF | LSC | 121 | 790 | 410 |  |  |
|  | clpP | LSC | 71 | 902 | 292 | 698 | 228 |
|  | ndhA | SSC | 552 | 1182 | 540 |  |  |
|  | ndhB | IR | 777 | 685 | 756 |  |  |
|  | petB | LSC | 6 | 802 | 642 |  |  |
|  | petD | LSC | 9 | 734 | 474 |  |  |
|  | rpl16 | LSC | 10 | 1073 | 398 |  |  |
| *Ficus* | rpl2 | IR | 390 | 685 | 435 |  |  |
| *tikoua* | rpoC1 | LSC | 435 | 794 | 1626 |  |  |
|  | rps12 | LSC | 114 |  | 232 | 536 | 26 |
|  | rps16 | LSC | 42 | 900 | 228 |  |  |
|  | trnA-UGC | IR | 38 | 802 | 35 |  |  |
|  | trnG-UCC | LSC | 23 | 721 | 48 |  |  |
|  | trnI-GAU | IR | 37 | 945 | 35 |  |  |
|  | trnK-UUU | LSC | 35 | 2597 | 37 |  |  |
|  | trnL-UAA | LSC | 37 | 500 | 50 |  |  |
|  | trnV-UAC | LSC | 35 | 619 | 39 |  |  |
|  | ycf3 | LSC | 126 | 790 | 228 | 751 | 153 |
|  | atpF | LSC | 119 | 783 | 412 |  |  |
|  | clpP | LSC | 71 | 893 | 292 | 699 | 228 |
|  | ndhA | SSC | 552 | 1173 | 540 |  |  |
|  | ndhB | IR | 777 | 685 | 756 |  |  |
|  | petB | LSC | 6 | 802 | 642 |  |  |
|  | petD | LSC | 9 | 734 | 474 |  |  |
|  | rpl16 | LSC | 10 | 1064 | 398 |  |  |
| *Ficus* | rpl2 | IR | 390 | 685 | 435 |  |  |
| *hispida* | rpoC1 | LSC | 435 | 806 | 1626 |  |  |
|  | rps12 | LSC | 114 |  | 232 | 536 | 26 |
|  | rps16 | LSC | 42 | 895 | 228 |  |  |
|  | trnA-UGC | IR | 38 | 802 | 35 |  |  |
|  | trnG-UCC | LSC | 23 | 722 | 48 |  |  |
|  | trnI-GAU | IR | 37 | 945 | 35 |  |  |
|  | trnK-UUU | LSC | 35 | 2593 | 37 |  |  |
|  | trnL-UAA | LSC | 35 | 507 | 50 |  |  |
|  | trnV-UAC | LSC | 37 | 618 | 37 |  |  |
|  | ycf3 | LSC | 124 | 789 | 228 | 745 | 155 |
|  | atpF | LSC | 121 | 782 | 410 |  |  |
|  | clpP | LSC | 71 | 903 | 292 | 697 | 263 |
|  | ndhA | SSC | 552 | 1182 | 540 |  |  |
|  | ndhB | IR | 777 | 685 | 756 |  |  |
|  | petB | LSC | 6 | 802 | 642 |  |  |
|  | petD | LSC | 9 | 741 | 474 |  |  |
|  | rpl16 | LSC | 10 | 1121 | 398 |  |  |
| *Ficus* | rpl2 | IR | 390 | 685 | 435 |  |  |
| *virens* | rpoC1 | LSC | 435 | 798 | 1626 |  |  |
|  | rps12 | LSC | 114 |  | 232 | 536 | 26 |
|  | rps16 | LSC | 42 | 900 | 228 |  |  |
|  | trnA-UGC | IR | 38 | 802 | 35 |  |  |
|  | trnG-UCC | LSC | 23 | 720 | 48 |  |  |
|  | trnI-GAU | IR | 37 | 945 | 35 |  |  |
|  | trnK-UUU | LSC | 35 | 2587 | 37 |  |  |
|  | trnL-UAA | LSC | 37 | 505 | 50 |  |  |
|  | trnV-UAC | LSC | 37 | 619 | 37 |  |  |
|  | ycf3 | LSC | 126 | 789 | 228 | 752 | 153 |
|  | atpF | LSC | 121 | 781 | 410 |  |  |
|  | clpP | LSC | 71 | 889 | 292 | 719 | 228 |
|  | ndhA | SSC | 552 | 1174 | 540 |  |  |
|  | ndhB | IR | 777 | 685 | 756 |  |  |
|  | petB | LSC | 6 | 819 | 642 |  |  |
|  | petD | LSC | 9 | 734 | 474 |  |  |
|  | rpl16 | LSC | 10 | 1063 | 398 |  |  |
| *Ficus* | rpl2 | IR | 390 | 685 | 435 |  |  |
| *sarmento* | rpoC1 | LSC | 435 | 807 | 1626 |  |  |
| *sa var.* | rps12 | LSC | 114 |  | 232 | 536 | 26 |
| *impressa* | rps16 | LSC | 42 | 903 | 228 |  |  |
|  | trnA-UGC | IR | 38 | 802 | 35 |  |  |
|  | trnG-UCC | LSC | 23 | 721 | 48 |  |  |
|  | trnI-GAU | IR | 37 | 945 | 35 |  |  |
|  | trnK-UUU | LSC | 35 | 2601 | 37 |  |  |
|  | trnL-UAA | LSC | 37 | 509 | 50 |  |  |
|  | trnV-UAC | LSC | 37 | 627 | 37 |  |  |
|  | ycf3 | LSC | 126 | 788 | 228 | 751 | 153 |
|  | atpF | LSC | 121 | 792 | 410 |  |  |
|  | clpP | LSC | 71 | 904 | 292 | 701 | 228 |
|  | ndhA | SSC | 552 | 1176 | 540 |  |  |
|  | ndhB | IR | 777 | 685 | 756 |  |  |
|  | petB | LSC | 6 | 802 | 642 |  |  |
|  | petD | LSC | 9 | 734 | 474 |  |  |
|  | rpl16 | LSC | 10 | 1077 | 398 |  |  |
| *Ficus* | rpl2 | IR | 390 | 685 | 435 |  |  |
| *pandurata* | rpoC1 | LSC | 435 | 800 | 1626 |  |  |
|  | rps12 | LSC | 114 |  | 232 | 536 | 26 |
|  | rps16 | LSC | 42 | 898 | 222 |  |  |
|  | trnA-UGC | IR | 38 | 802 | 35 |  |  |
|  | trnG-UCC | LSC | 23 | 721 | 48 |  |  |
|  | trnI-GAU | IR | 37 | 945 | 35 |  |  |
|  | trnK-UUU | LSC | 35 | 2593 | 37 |  |  |
|  | trnL-UAA | LSC | 37 | 505 | 50 |  |  |
|  | trnV-UAC | LSC | 35 | 618 | 39 |  |  |
|  | ycf3 | LSC | 126 | 787 | 228 | 751 | 153 |
|  | atpF | LSC | 121 | 744 | 410 |  |  |
|  | clpP | LSC | 71 | 903 | 292 | 701 | 228 |
|  | ndhA | SSC | 552 | 1131 | 540 |  |  |
|  | ndhB | IR | 777 | 685 | 756 |  |  |
|  | petB | LSC | 6 | 802 | 642 |  |  |
|  | petD | LSC | 9 | 734 | 474 |  |  |
|  | rpl16 | LSC | 10 | 1097 | 398 |  |  |
| *Ficus* | rpl2 | IR | 390 | 685 | 435 |  |  |
| *microcarpa* | rpoC1 | LSC | 435 | 795 | 1626 |  |  |
|  | rps12 | LSC | 114 |  | 232 | 536 | 26 |
|  | rps16 | LSC | 42 | 899 | 228 |  |  |
|  | trnA-UGC | IR | 38 | 802 | 35 |  |  |
|  | trnG-UCC | LSC | 23 | 721 | 48 |  |  |
|  | trnI-GAU | IR | 37 | 945 | 35 |  |  |
|  | trnK-UUU | LSC | 35 | 2587 | 37 |  |  |
|  | trnL-UAA | LSC | 37 | 505 | 50 |  |  |
|  | trnV-UAC | LSC | 37 | 613 | 37 |  |  |
|  | ycf3 | LSC | 126 | 771 | 228 | 752 | 153 |
|  | atpF | LSC | 121 | 783 | 410 |  |  |
|  | clpP | LSC | 71 | 903 | 292 | 698 | 228 |
|  | ndhA | SSC | 552 | 1175 | 540 |  |  |
|  | ndhB | IR | 777 | 685 | 756 |  |  |
|  | petB | LSC | 6 | 802 | 642 |  |  |
|  | petD | LSC | 9 | 734 | 474 |  |  |
|  | rpl16 | LSC | 10 | 1045 | 398 |  |  |
| *Ficus* | rpl2 | IR | 390 | 685 | 435 |  |  |
| *formosana* | rpoC1 | LSC | 435 | 795 | 1626 |  |  |
|  | rps12 | LSC | 114 |  | 232 | 536 | 26 |
|  | rps16 | LSC | 42 | 894 | 228 |  |  |
|  | trnA-UGC | IR | 38 | 802 | 35 |  |  |
|  | trnG-UCC | LSC | 23 | 721 | 48 |  |  |
|  | trnI-GAU | IR | 37 | 945 | 35 |  |  |
|  | trnK-UUU | LSC | 35 | 2588 | 37 |  |  |
|  | trnL-UAA | LSC | 37 | 505 | 50 |  |  |
|  | trnV-UAC | LSC | 37 | 618 | 37 |  |  |
|  | ycf3 | LSC | 124 | 789 | 230 | 751 | 153 |
|  | atpF | LSC | 121 | 792 | 410 |  |  |
|  | clpP | LSC | 71 | 907 | 295 | 679 | 228 |
|  | ndhA | SSC | 552 | 1177 | 540 |  |  |
|  | ndhB | IR | 777 | 685 | 756 |  |  |
|  | petB | LSC | 6 | 802 | 642 |  |  |
|  | petD | LSC | 9 | 734 | 474 |  |  |
|  | rpl16 | LSC | 10 | 1074 | 398 |  |  |
| *Ficus* | rpl2 | IR | 390 | 685 | 435 |  |  |
| *sarmentosa* | rpoC1 | LSC | 435 | 799 | 1626 |  |  |
| *var.* | rps12 | LSC | 114 |  | 232 | 536 | 26 |
| *lacrymans* | rps16 | LSC | 42 | 896 | 228 |  |  |
|  | trnA-UGC | IR | 38 | 802 | 35 |  |  |
|  | trnG-UCC | LSC | 23 | 721 | 48 |  |  |
|  | trnI-GAU | IR | 37 | 945 | 35 |  |  |
|  | trnK-UUU | LSC | 35 | 2593 | 38 |  |  |
|  | trnL-UAA | LSC | 37 | 505 | 50 |  |  |
|  | trnV-UAC | LSC | 37 | 618 | 37 |  |  |
|  | ycf3 | LSC | 124 | 795 | 230 | 751 | 153 |
|  | atpF | LSC | 121 | 783 | 410 |  |  |
|  | clpP | LSC | 71 | 894 | 292 | 698 | 228 |
|  | ndhA | SSC | 552 | 1175 | 540 |  |  |
|  | ndhB | IR | 777 | 685 | 756 |  |  |
|  | petB | LSC | 6 | 802 | 642 |  |  |
|  | petD | LSC | 9 | 734 | 474 |  |  |
|  | rpl16 | LSC | 10 | 1072 | 398 |  |  |
| *Ficus* | rpl2 | IR | 390 | 685 | 435 |  |  |
| *simpliciss* | rpoC1 | LSC | 435 | 794 | 1626 |  |  |
| *ima* | rps12 | LSC | 114 |  | 232 | 536 | 26 |
|  | rps16 | LSC | 42 | 893 | 228 |  |  |
|  | trnA-UGC | IR | 38 | 802 | 35 |  |  |
|  | trnG-UCC | LSC | 23 | 720 | 48 |  |  |
|  | trnI-GAU | IR | 37 | 945 | 35 |  |  |
|  | trnK-UUU | LSC | 35 | 2596 | 38 |  |  |
|  | trnL-UAA | LSC | 37 | 505 | 50 |  |  |
|  | trnV-UAC | LSC | 37 | 618 | 37 |  |  |
|  | ycf3 |  | 124 | 789 | 230 | 751 | 153 |
|  | atpF | LSC | 121 | 790 | 410 |  |  |
|  | clpP | LSC | 71 | 753 | 292 | 701 | 216 |
|  | ndhA | SSC | 552 | 1183 | 540 |  |  |
|  | ndhB | IR | 777 | 685 | 756 |  |  |
|  | petB | LSC | 6 | 802 | 642 |  |  |
|  | petD | LSC | 9 | 729 | 474 |  |  |
|  | rpl16 | LSC | 10 | 1070 | 398 |  |  |
| *Ficus* | rpl2 | IR | 390 | 685 | 435 |  |  |
| *tinctoria* | rpoC1 | LSC | 435 | 793 | 1626 |  |  |
|  | rps12 | LSC | 114 |  | 232 | 543 | 26 |
|  | rps16 | LSC | 42 | 892 | 228 |  |  |
|  | trnA-UGC | IR | 38 | 802 | 35 |  |  |
|  | trnG-UCC | LSC | 23 | 721 | 48 |  |  |
|  | trnI-GAU | IR | 37 | 946 | 35 |  |  |
|  | trnK-UUU | LSC | 35 | 2583 | 37 |  |  |
|  | trnL-UAA | LSC | 37 | 505 | 50 |  |  |
|  | trnV-UAC | LSC | 37 | 618 | 37 |  |  |
|  | ycf3 | LSC | 124 | 786 | 230 | 751 | 153 |
