# Supplementary material for: Comprehensive analysis of complete chloroplast genome and phylogenetic aspects of ten Ficus species
Source: BMC Plant Biol. 2022 May 23;22:253. doi: 10.1186/s12870-022-03643-4 (PMC9125854; doi:10.1186/s12870-022-03643-4)
Supplement: Supplementary file 5 — Additional file 5: Table S4. Codon usage of the eleven Ficus chloroplast genomes. [file 12870_2022_3643_MOESM5_ESM.doc]

**Table S4**. Codon usage of the eleven *Ficus* chloroplast genomes.

| *Ficsu pumila* | | | | | | | |
| --- | --- | --- | --- | --- | --- | --- | --- |
| Amino acid | Codon | Count | RSCU | Amino acid | Codon | Count | RSCU |
| Phe | UUU(F) | 2383 | 1.27 | Tyr | UAU(Y) | 1631 | 1.39 |
| Phe | UUC(F) | 1356 | 0.73 | Tyr | UAC(Y) | 713 | 0.61 |
| Leu | UUA(L) | 1348 | 1.49 | Stop | UAA(*) | 1341 | 1.28 |
| Leu | UUG(L) | 1035 | 1.14 | Stop | UAG(*) | 749 | 0.72 |
| Leu | CUU(L) | 1120 | 1.24 | His | CAU(H) | 953 | 1.42 |
| Leu | CUC(L) | 635 | 0.7 | His | CAC(H) | 391 | 0.58 |
| Leu | CUA(L) | 814 | 0.9 | Gln | CAA(Q) | 981 | 1.39 |
| Leu | CUG(L) | 484 | 0.53 | Gln | CAG(Q) | 426 | 0.61 |
| Ile | AUU(I) | 1896 | 1.2 | Asn | AAU(N) | 2027 | 1.44 |
| Ile | AUC(I) | 1079 | 0.68 | Asn | AAC(N) | 782 | 0.56 |
| Ile | AUA(I) | 1759 | 1.11 | Lys | AAA(K) | 2266 | 1.37 |
| Met | AUG(M) | 912 | 1 | Lys | AAG(K) | 1045 | 0.63 |
| Val | GUU(V) | 790 | 1.34 | Asp | GAU(D) | 1105 | 1.46 |
| Val | GUC(V) | 408 | 0.69 | Asp | GAC(D) | 410 | 0.54 |
| Val | GUA(V) | 750 | 1.27 | Glu | GAA(E) | 1304 | 1.4 |
| Val | GUG(V) | 417 | 0.71 | Glu | GAG(E) | 557 | 0.6 |
| Ser | UCU(S) | 1286 | 1.55 | Cys | UGU(C) | 738 | 1.23 |
| Ser | UCC(S) | 829 | 1 | Cys | UGC(C) | 462 | 0.77 |
| Ser | UCA(S) | 1057 | 1.27 | Stop | UGA(*) | 1047 | 1 |
| Ser | UCG(S) | 565 | 0.68 | Trp | UGG(W) | 652 | 1 |
| Pro | CCU(P) | 693 | 1.18 | Arg | CGU(R) | 389 | 0.72 |
| Pro | CCC(P) | 557 | 0.95 | Arg | CGC(R) | 232 | 0.43 |
| Pro | CCA(P) | 728 | 1.24 | Arg | CGA(R) | 542 | 1 |
| Pro | CCG(P) | 372 | 0.63 | Arg | CGG(R) | 374 | 0.69 |
| Thr | ACU(T) | 707 | 1.22 | Ser | AGU(S) | 753 | 0.9 |
| Thr | ACC(T) | 559 | 0.97 | Ser | AGC(S) | 503 | 0.6 |
| Thr | ACA(T) | 708 | 1.22 | Arg | AGA(R) | 1099 | 2.03 |
| Thr | ACG(T) | 338 | 0.58 | Arg | AGG(R) | 615 | 1.14 |
| Ala | GCU(A) | 517 | 1.34 | Gly | GGU(G) | 581 | 1.05 |
| Ala | GCC(A) | 379 | 0.98 | Gly | GGC(G) | 365 | 0.66 |
| Ala | GCA(A) | 447 | 1.16 | Gly | GGA(G) | 746 | 1.35 |
| Ala | GCG(A) | 200 | 0.52 | Gly | GGG(G) | 519 | 0.94 |
| *Ficsu tikoua* | | | | | | | |
| Phe | UUU(F) | 2452 | 1.26 | Tyr | UAU(Y) | 1678 | 1.38 |
| Phe | UUC(F) | 1430 | 0.74 | Tyr | UAC(Y) | 761 | 0.62 |
| Leu | UUA(L) | 1271 | 1.4 | Stop | UAA(*) | 1458 | 1.33 |
| Leu | UUG(L) | 1146 | 1.26 | Stop | UAG(*) | 817 | 0.74 |
| Leu | CUU(L) | 1081 | 1.19 | His | CAU(H) | 915 | 1.4 |
| Leu | CUC(L) | 682 | 0.75 | His | CAC(H) | 394 | 0.6 |
| Leu | CUA(L) | 803 | 0.88 | Gln | CAA(Q) | 1089 | 1.44 |
| Leu | CUG(L) | 462 | 0.51 | Gln | CAG(Q) | 420 | 0.56 |
| Ile | AUU(I) | 1860 | 1.18 | Asn | AAU(N) | 1972 | 1.42 |
| Ile | AUC(I) | 1097 | 0.7 | Asn | AAC(N) | 802 | 0.58 |
| Ile | AUA(I) | 1770 | 1.12 | Lys | AAA(K) | 2267 | 1.36 |
| Met | AUG(M) | 902 | 1 | Lys | AAG(K) | 1069 | 0.64 |
| Val | GUU(V) | 811 | 1.37 | Asp | GAU(D) | 1099 | 1.44 |
| Val | GUC(V) | 444 | 0.75 | Asp | GAC(D) | 432 | 0.56 |
| Val | GUA(V) | 723 | 1.22 | Glu | GAA(E) | 1314 | 1.38 |
| Val | GUG(V) | 398 | 0.67 | Glu | GAG(E) | 594 | 0.62 |
| Ser | UCU(S) | 1244 | 1.51 | Cys | UGU(C) | 707 | 1.21 |
| Ser | UCC(S) | 883 | 1.07 | Cys | UGC(C) | 459 | 0.79 |
| Ser | UCA(S) | 1022 | 1.24 | Stop | UGA(*) | 1025 | 0.93 |
| Ser | UCG(S) | 597 | 0.72 | Trp | UGG(W) | 682 | 1 |
| Pro | CCU(P) | 599 | 1.04 | Arg | CGU(R) | 385 | 0.71 |
| Pro | CCC(P) | 588 | 1.03 | Arg | CGC(R) | 241 | 0.45 |
| Pro | CCA(P) | 723 | 1.26 | Arg | CGA(R) | 556 | 1.03 |
| Pro | CCG(P) | 384 | 0.67 | Arg | CGG(R) | 360 | 0.67 |
| Thr | ACU(T) | 691 | 1.23 | Ser | AGU(S) | 707 | 0.86 |
| Thr | ACC(T) | 527 | 0.94 | Ser | AGC(S) | 499 | 0.6 |
| Thr | ACA(T) | 676 | 1.21 | Arg | AGA(R) | 1123 | 2.07 |
| Thr | ACG(T) | 349 | 0.62 | Arg | AGG(R) | 583 | 1.08 |
| Ala | GCU(A) | 439 | 1.24 | Gly | GGU(G) | 510 | 0.96 |
| Ala | GCC(A) | 353 | 1 | Gly | GGC(G) | 339 | 0.64 |
| Ala | GCA(A) | 387 | 1.09 | Gly | GGA(G) | 746 | 1.4 |
| Ala | GCG(A) | 239 | 0.67 | Gly | GGG(G) | 530 | 1 |
| *Ficus hispida* | | | | | | | |
| Phe | UUU(F) | 2347 | 1.23 | Tyr | UAU(Y) | 1643 | 1.39 |
| Phe | UUC(F) | 1474 | 0.77 | Tyr | UAC(Y) | 718 | 0.61 |
| Leu | UUA(L) | 1302 | 1.46 | Stop | UAA(*) | 1354 | 1.34 |
| Leu | UUG(L) | 1087 | 1.22 | Stop | UAG(*) | 721 | 0.71 |
| Leu | CUU(L) | 1112 | 1.24 | His | CAU(H) | 946 | 1.45 |
| Leu | CUC(L) | 666 | 0.75 | His | CAC(H) | 360 | 0.55 |
| Leu | CUA(L) | 725 | 0.81 | Gln | CAA(Q) | 1047 | 1.43 |
| Leu | CUG(L) | 469 | 0.52 | Gln | CAG(Q) | 418 | 0.57 |
| Ile | AUU(I) | 1961 | 1.2 | Asn | AAU(N) | 2048 | 1.45 |
| Ile | AUC(I) | 1166 | 0.71 | Asn | AAC(N) | 784 | 0.55 |
| Ile | AUA(I) | 1777 | 1.09 | Lys | AAA(K) | 2294 | 1.37 |
| Met | AUG(M) | 885 | 1 | Lys | AAG(K) | 1047 | 0.63 |
| Val | GUU(V) | 833 | 1.39 | Asp | GAU(D) | 1111 | 1.48 |
| Val | GUC(V) | 410 | 0.68 | Asp | GAC(D) | 387 | 0.52 |
| Val | GUA(V) | 711 | 1.18 | Glu | GAA(E) | 1289 | 1.38 |
| Val | GUG(V) | 449 | 0.75 | Glu | GAG(E) | 581 | 0.62 |
| Ser | UCU(S) | 1263 | 1.55 | Cys | UGU(C) | 738 | 1.25 |
| Ser | UCC(S) | 855 | 1.05 | Cys | UGC(C) | 440 | 0.75 |
| Ser | UCA(S) | 887 | 1.09 | Stop | UGA(*) | 963 | 0.95 |
| Ser | UCG(S) | 614 | 0.75 | Trp | UGG(W) | 671 | 1 |
| Pro | CCU(P) | 668 | 1.15 | Arg | CGU(R) | 368 | 0.68 |
| Pro | CCC(P) | 540 | 0.93 | Arg | CGC(R) | 232 | 0.43 |
| Pro | CCA(P) | 721 | 1.24 | Arg | CGA(R) | 555 | 1.03 |
| Pro | CCG(P) | 392 | 0.68 | Arg | CGG(R) | 350 | 0.65 |
| Thr | ACU(T) | 732 | 1.2 | Ser | AGU(S) | 733 | 0.9 |
| Thr | ACC(T) | 593 | 0.97 | Ser | AGC(S) | 551 | 0.67 |
| Thr | ACA(T) | 698 | 1.15 | Arg | AGA(R) | 1104 | 2.05 |
| Thr | ACG(T) | 410 | 0.67 | Arg | AGG(R) | 630 | 1.17 |
| Ala | GCU(A) | 491 | 1.32 | Gly | GGU(G) | 531 | 1 |
| Ala | GCC(A) | 341 | 0.92 | Gly | GGC(G) | 327 | 0.62 |
| Ala | GCA(A) | 425 | 1.14 | Gly | GGA(G) | 745 | 1.41 |
| Ala | GCG(A) | 233 | 0.63 | Gly | GGG(G) | 517 | 0.98 |
| *Ficus virens* | | | | | | | |
| Phe | UUU(F) | 2317 | 1.23 | Tyr | UAU(Y) | 1705 | 1.41 |
| Phe | UUC(F) | 1437 | 0.77 | Tyr | UAC(Y) | 713 | 0.59 |
| Leu | UUA(L) | 1325 | 1.46 | Stop | UAA(*) | 1311 | 1.3 |
| Leu | UUG(L) | 1140 | 1.26 | Stop | UAG(*) | 748 | 0.74 |
| Leu | CUU(L) | 1125 | 1.24 | His | CAU(H) | 1002 | 1.45 |
| Leu | CUC(L) | 631 | 0.7 | His | CAC(H) | 381 | 0.55 |
| Leu | CUA(L) | 759 | 0.84 | Gln | CAA(Q) | 1018 | 1.39 |
| Leu | CUG(L) | 458 | 0.51 | Gln | CAG(Q) | 452 | 0.61 |
| Ile | AUU(I) | 1967 | 1.24 | Asn | AAU(N) | 2019 | 1.45 |
| Ile | AUC(I) | 1058 | 0.66 | Asn | AAC(N) | 765 | 0.55 |
| Ile | AUA(I) | 1753 | 1.1 | Lys | AAA(K) | 2315 | 1.37 |
| Met | AUG(M) | 853 | 1 | Lys | AAG(K) | 1061 | 0.63 |
| Val | GUU(V) | 853 | 1.4 | Asp | GAU(D) | 1175 | 1.49 |
| Val | GUC(V) | 417 | 0.68 | Asp | GAC(D) | 403 | 0.51 |
| Val | GUA(V) | 775 | 1.27 | Glu | GAA(E) | 1365 | 1.4 |
| Val | GUG(V) | 400 | 0.65 | Glu | GAG(E) | 587 | 0.6 |
| Ser | UCU(S) | 1253 | 1.56 | Cys | UGU(C) | 708 | 1.24 |
| Ser | UCC(S) | 853 | 1.06 | Cys | UGC(C) | 437 | 0.76 |
| Ser | UCA(S) | 952 | 1.18 | Stop | UGA(*) | 959 | 0.95 |
| Ser | UCG(S) | 586 | 0.73 | Trp | UGG(W) | 675 | 1 |
| Pro | CCU(P) | 694 | 1.16 | Arg | CGU(R) | 374 | 0.7 |
| Pro | CCC(P) | 556 | 0.93 | Arg | CGC(R) | 238 | 0.44 |
| Pro | CCA(P) | 772 | 1.29 | Arg | CGA(R) | 564 | 1.05 |
| Pro | CCG(P) | 368 | 0.62 | Arg | CGG(R) | 353 | 0.66 |
| Thr | ACU(T) | 719 | 1.21 | Ser | AGU(S) | 696 | 0.86 |
| Thr | ACC(T) | 601 | 1.01 | Ser | AGC(S) | 490 | 0.61 |
| Thr | ACA(T) | 683 | 1.15 | Arg | AGA(R) | 1050 | 1.96 |
| Thr | ACG(T) | 373 | 0.63 | Arg | AGG(R) | 635 | 1.19 |
| Ala | GCU(A) | 487 | 1.3 | Gly | GGU(G) | 551 | 1.04 |
| Ala | GCC(A) | 333 | 0.89 | Gly | GGC(G) | 328 | 0.62 |
| Ala | GCA(A) | 447 | 1.2 | Gly | GGA(G) | 751 | 1.41 |
| Ala | GCG(A) | 227 | 0.61 | Gly | GGG(G) | 499 | 0.94 |
| *Ficus sarmentosa var. impressa* | | | | | | | |
| Phe | UUU(F) | 2316 | 1.23 | Tyr | UAU(Y) | 1711 | 1.42 |
| Phe | UUC(F) | 1444 | 0.77 | Tyr | UAC(Y) | 707 | 0.58 |
| Leu | UUA(L) | 1330 | 1.45 | Stop | UAA(*) | 1426 | 1.31 |
| Leu | UUG(L) | 1069 | 1.17 | Stop | UAG(*) | 810 | 0.75 |
| Leu | CUU(L) | 1116 | 1.22 | His | CAU(H) | 956 | 1.46 |
| Leu | CUC(L) | 654 | 0.71 | His | CAC(H) | 352 | 0.54 |
| Leu | CUA(L) | 841 | 0.92 | Gln | CAA(Q) | 1064 | 1.43 |
| Leu | CUG(L) | 479 | 0.52 | Gln | CAG(Q) | 426 | 0.57 |
| Ile | AUU(I) | 1919 | 1.22 | Asn | AAU(N) | 1849 | 1.43 |
| Ile | AUC(I) | 1095 | 0.7 | Asn | AAC(N) | 737 | 0.57 |
| Ile | AUA(I) | 1693 | 1.08 | Lys | AAA(K) | 2287 | 1.37 |
| Met | AUG(M) | 780 | 1 | Lys | AAG(K) | 1042 | 0.63 |
| Val | GUU(V) | 836 | 1.38 | Asp | GAU(D) | 1136 | 1.44 |
| Val | GUC(V) | 425 | 0.7 | Asp | GAC(D) | 447 | 0.56 |
| Val | GUA(V) | 783 | 1.29 | Glu | GAA(E) | 1358 | 1.36 |
| Val | GUG(V) | 376 | 0.62 | Glu | GAG(E) | 632 | 0.64 |
| Ser | UCU(S) | 1290 | 1.61 | Cys | UGU(C) | 736 | 1.24 |
| Ser | UCC(S) | 776 | 0.97 | Cys | UGC(C) | 451 | 0.76 |
| Ser | UCA(S) | 1073 | 1.34 | Stop | UGA(*) | 1023 | 0.94 |
| Ser | UCG(S) | 569 | 0.71 | Trp | UGG(W) | 691 | 1 |
| Pro | CCU(P) | 696 | 1.17 | Arg | CGU(R) | 420 | 0.76 |
| Pro | CCC(P) | 535 | 0.9 | Arg | CGC(R) | 228 | 0.41 |
| Pro | CCA(P) | 779 | 1.31 | Arg | CGA(R) | 575 | 1.05 |
| Pro | CCG(P) | 371 | 0.62 | Arg | CGG(R) | 386 | 0.7 |
| Thr | ACU(T) | 709 | 1.22 | Ser | AGU(S) | 664 | 0.83 |
| Thr | ACC(T) | 569 | 0.98 | Ser | AGC(S) | 431 | 0.54 |
| Thr | ACA(T) | 709 | 1.22 | Arg | AGA(R) | 1085 | 1.97 |
| Thr | ACG(T) | 345 | 0.59 | Arg | AGG(R) | 606 | 1.1 |
| Ala | GCU(A) | 481 | 1.27 | Gly | GGU(G) | 542 | 1.01 |
| Ala | GCC(A) | 353 | 0.93 | Gly | GGC(G) | 319 | 0.59 |
| Ala | GCA(A) | 444 | 1.17 | Gly | GGA(G) | 781 | 1.45 |
| Ala | GCG(A) | 238 | 0.63 | Gly | GGG(G) | 511 | 0.95 |
| *Ficus pandurata* | | | | | | | |
| Phe | UUU(F) | 2407 | 1.22 | Tyr | UAU(Y) | 1697 | 1.38 |
| Phe | UUC(F) | 1536 | 0.78 | Tyr | UAC(Y) | 754 | 0.62 |
| Leu | UUA(L) | 1264 | 1.37 | Stop | UAA(*) | 1352 | 1.32 |
| Leu | UUG(L) | 1160 | 1.25 | Stop | UAG(*) | 788 | 0.77 |
| Leu | CUU(L) | 1132 | 1.22 | His | CAU(H) | 966 | 1.41 |
| Leu | CUC(L) | 641 | 0.69 | His | CAC(H) | 400 | 0.59 |
| Leu | CUA(L) | 825 | 0.89 | Gln | CAA(Q) | 1141 | 1.42 |
| Leu | CUG(L) | 525 | 0.57 | Gln | CAG(Q) | 466 | 0.58 |
| Ile | AUU(I) | 1977 | 1.2 | Asn | AAU(N) | 1974 | 1.43 |
| Ile | AUC(I) | 1114 | 0.68 | Asn | AAC(N) | 786 | 0.57 |
| Ile | AUA(I) | 1840 | 1.12 | Lys | AAA(K) | 2279 | 1.34 |
| Met | AUG(M) | 925 | 1 | Lys | AAG(K) | 1117 | 0.66 |
| Val | GUU(V) | 782 | 1.32 | Asp | GAU(D) | 1045 | 1.43 |
| Val | GUC(V) | 452 | 0.76 | Asp | GAC(D) | 414 | 0.57 |
| Val | GUA(V) | 718 | 1.21 | Glu | GAA(E) | 1334 | 1.39 |
| Val | GUG(V) | 412 | 0.7 | Glu | GAG(E) | 587 | 0.61 |
| Ser | UCU(S) | 1196 | 1.52 | Cys | UGU(C) | 671 | 1.23 |
| Ser | UCC(S) | 873 | 1.11 | Cys | UGC(C) | 417 | 0.77 |
| Ser | UCA(S) | 827 | 1.05 | Stop | UGA(*) | 932 | 0.91 |
| Ser | UCG(S) | 609 | 0.77 | Trp | UGG(W) | 659 | 1 |
| Pro | CCU(P) | 629 | 1.11 | Arg | CGU(R) | 383 | 0.72 |
| Pro | CCC(P) | 557 | 0.98 | Arg | CGC(R) | 240 | 0.45 |
| Pro | CCA(P) | 728 | 1.28 | Arg | CGA(R) | 582 | 1.1 |
| Pro | CCG(P) | 361 | 0.63 | Arg | CGG(R) | 348 | 0.65 |
| Thr | ACU(T) | 664 | 1.18 | Ser | AGU(S) | 732 | 0.93 |
| Thr | ACC(T) | 564 | 1 | Ser | AGC(S) | 478 | 0.61 |
| Thr | ACA(T) | 656 | 1.17 | Arg | AGA(R) | 1038 | 1.95 |
| Thr | ACG(T) | 366 | 0.65 | Arg | AGG(R) | 598 | 1.13 |
| Ala | GCU(A) | 485 | 1.34 | Gly | GGU(G) | 547 | 1 |
| Ala | GCC(A) | 344 | 0.95 | Gly | GGC(G) | 320 | 0.59 |
| Ala | GCA(A) | 384 | 1.06 | Gly | GGA(G) | 765 | 1.4 |
| Ala | GCG(A) | 238 | 0.66 | Gly | GGG(G) | 547 | 1 |
| *Ficus microcarpa* | | | | | | | |
| Phe | UUU(F) | 2391 | 1.25 | Tyr | UAU(Y) | 1708 | 1.41 |
| Phe | UUC(F) | 1448 | 0.75 | Tyr | UAC(Y) | 723 | 0.59 |
| Leu | UUA(L) | 1353 | 1.5 | Stop | UAA(*) | 1347 | 1.28 |
| Leu | UUG(L) | 1112 | 1.23 | Stop | UAG(*) | 786 | 0.75 |
| Leu | CUU(L) | 1074 | 1.19 | His | CAU(H) | 897 | 1.46 |
| Leu | CUC(L) | 600 | 0.66 | His | CAC(H) | 329 | 0.54 |
| Leu | CUA(L) | 785 | 0.87 | Gln | CAA(Q) | 1060 | 1.44 |
| Leu | CUG(L) | 494 | 0.55 | Gln | CAG(Q) | 412 | 0.56 |
| Ile | AUU(I) | 1861 | 1.19 | Asn | AAU(N) | 1909 | 1.43 |
| Ile | AUC(I) | 1096 | 0.7 | Asn | AAC(N) | 754 | 0.57 |
| Ile | AUA(I) | 1727 | 1.11 | Lys | AAA(K) | 2379 | 1.39 |
| Met | AUG(M) | 838 | 1 | Lys | AAG(K) | 1047 | 0.61 |
| Val | GUU(V) | 821 | 1.37 | Asp | GAU(D) | 1090 | 1.46 |
| Val | GUC(V) | 428 | 0.71 | Asp | GAC(D) | 402 | 0.54 |
| Val | GUA(V) | 755 | 1.26 | Glu | GAA(E) | 1412 | 1.41 |
| Val | GUG(V) | 396 | 0.66 | Glu | GAG(E) | 585 | 0.59 |
| Ser | UCU(S) | 1231 | 1.53 | Cys | UGU(C) | 697 | 1.24 |
| Ser | UCC(S) | 878 | 1.09 | Cys | UGC(C) | 430 | 0.76 |
| Ser | UCA(S) | 999 | 1.24 | Stop | UGA(*) | 1031 | 0.98 |
| Ser | UCG(S) | 608 | 0.75 | Trp | UGG(W) | 650 | 1 |
| Pro | CCU(P) | 707 | 1.18 | Arg | CGU(R) | 407 | 0.78 |
| Pro | CCC(P) | 568 | 0.95 | Arg | CGC(R) | 237 | 0.46 |
| Pro | CCA(P) | 752 | 1.25 | Arg | CGA(R) | 569 | 1.1 |
| Pro | CCG(P) | 377 | 0.63 | Arg | CGG(R) | 311 | 0.6 |
| Thr | ACU(T) | 716 | 1.21 | Ser | AGU(S) | 695 | 0.86 |
| Thr | ACC(T) | 579 | 0.98 | Ser | AGC(S) | 426 | 0.53 |
| Thr | ACA(T) | 699 | 1.18 | Arg | AGA(R) | 1026 | 1.97 |
| Thr | ACG(T) | 379 | 0.64 | Arg | AGG(R) | 567 | 1.09 |
| Ala | GCU(A) | 565 | 1.39 | Gly | GGU(G) | 604 | 1.09 |
| Ala | GCC(A) | 332 | 0.82 | Gly | GGC(G) | 312 | 0.56 |
| Ala | GCA(A) | 483 | 1.19 | Gly | GGA(G) | 810 | 1.46 |
| Ala | GCG(A) | 249 | 0.61 | Gly | GGG(G) | 499 | 0.9 |
| *Ficus formosana* | | | | | | | |
| Phe | UUU(F) | 2367 | 1.25 | Tyr | UAU(Y) | 1683 | 1.41 |
| Phe | UUC(F) | 1425 | 0.75 | Tyr | UAC(Y) | 711 | 0.59 |
| Leu | UUA(L) | 1291 | 1.4 | Stop | UAA(*) | 1352 | 1.28 |
| Leu | UUG(L) | 1159 | 1.25 | Stop | UAG(*) | 799 | 0.76 |
| Leu | CUU(L) | 1146 | 1.24 | His | CAU(H) | 942 | 1.42 |
| Leu | CUC(L) | 671 | 0.73 | His | CAC(H) | 381 | 0.58 |
| Leu | CUA(L) | 787 | 0.85 | Gln | CAA(Q) | 1039 | 1.37 |
| Leu | CUG(L) | 498 | 0.54 | Gln | CAG(Q) | 481 | 0.63 |
| Ile | AUU(I) | 1966 | 1.22 | Asn | AAU(N) | 2004 | 1.45 |
| Ile | AUC(I) | 1072 | 0.67 | Asn | AAC(N) | 767 | 0.55 |
| Ile | AUA(I) | 1790 | 1.11 | Lys | AAA(K) | 2299 | 1.34 |
| Met | AUG(M) | 882 | 1 | Lys | AAG(K) | 1127 | 0.66 |
| Val | GUU(V) | 831 | 1.4 | Asp | GAU(D) | 1071 | 1.44 |
| Val | GUC(V) | 410 | 0.69 | Asp | GAC(D) | 418 | 0.56 |
| Val | GUA(V) | 727 | 1.23 | Glu | GAA(E) | 1250 | 1.32 |
| Val | GUG(V) | 405 | 0.68 | Glu | GAG(E) | 638 | 0.68 |
| Ser | UCU(S) | 1230 | 1.52 | Cys | UGU(C) | 700 | 1.2 |
| Ser | UCC(S) | 873 | 1.08 | Cys | UGC(C) | 462 | 0.8 |
| Ser | UCA(S) | 975 | 1.21 | Stop | UGA(*) | 1013 | 0.96 |
| Ser | UCG(S) | 570 | 0.71 | Trp | UGG(W) | 683 | 1 |
| Pro | CCU(P) | 659 | 1.09 | Arg | CGU(R) | 362 | 0.67 |
| Pro | CCC(P) | 542 | 0.9 | Arg | CGC(R) | 249 | 0.46 |
| Pro | CCA(P) | 804 | 1.33 | Arg | CGA(R) | 562 | 1.04 |
| Pro | CCG(P) | 408 | 0.68 | Arg | CGG(R) | 377 | 0.7 |
| Thr | ACU(T) | 709 | 1.23 | Ser | AGU(S) | 710 | 0.88 |
| Thr | ACC(T) | 528 | 0.92 | Ser | AGC(S) | 487 | 0.6 |
| Thr | ACA(T) | 673 | 1.17 | Arg | AGA(R) | 1089 | 2.02 |
| Thr | ACG(T) | 394 | 0.68 | Arg | AGG(R) | 595 | 1.1 |
| Ala | GCU(A) | 435 | 1.27 | Gly | GGU(G) | 501 | 0.96 |
| Ala | GCC(A) | 320 | 0.94 | Gly | GGC(G) | 326 | 0.63 |
| Ala | GCA(A) | 397 | 1.16 | Gly | GGA(G) | 714 | 1.37 |
| Ala | GCG(A) | 213 | 0.62 | Gly | GGG(G) | 538 | 1.04 |
| *Ficus sarmentosa var. lacrymans* | | | | | | | |
| Phe | UUU(F) | 2366 | 1.23 | Tyr | UAU(Y) | 1701 | 1.4 |
| Phe | UUC(F) | 1486 | 0.77 | Tyr | UAC(Y) | 731 | 0.6 |
| Leu | UUA(L) | 1236 | 1.37 | Stop | UAA(*) | 1362 | 1.28 |
| Leu | UUG(L) | 1128 | 1.25 | Stop | UAG(*) | 803 | 0.76 |
| Leu | CUU(L) | 1147 | 1.28 | His | CAU(H) | 940 | 1.42 |
| Leu | CUC(L) | 634 | 0.7 | His | CAC(H) | 381 | 0.58 |
| Leu | CUA(L) | 750 | 0.83 | Gln | CAA(Q) | 1067 | 1.4 |
| Leu | CUG(L) | 502 | 0.56 | Gln | CAG(Q) | 458 | 0.6 |
| Ile | AUU(I) | 1997 | 1.23 | Asn | AAU(N) | 1972 | 1.44 |
| Ile | AUC(I) | 1088 | 0.67 | Asn | AAC(N) | 766 | 0.56 |
| Ile | AUA(I) | 1773 | 1.09 | Lys | AAA(K) | 2281 | 1.33 |
| Met | AUG(M) | 858 | 1 | Lys | AAG(K) | 1145 | 0.67 |
| Val | GUU(V) | 841 | 1.4 | Asp | GAU(D) | 1069 | 1.43 |
| Val | GUC(V) | 426 | 0.71 | Asp | GAC(D) | 426 | 0.57 |
| Val | GUA(V) | 733 | 1.22 | Glu | GAA(E) | 1330 | 1.39 |
| Val | GUG(V) | 396 | 0.66 | Glu | GAG(E) | 586 | 0.61 |
| Ser | UCU(S) | 1199 | 1.51 | Cys | UGU(C) | 668 | 1.19 |
| Ser | UCC(S) | 898 | 1.13 | Cys | UGC(C) | 453 | 0.81 |
| Ser | UCA(S) | 895 | 1.13 | Stop | UGA(*) | 1017 | 0.96 |
| Ser | UCG(S) | 602 | 0.76 | Trp | UGG(W) | 663 | 1 |
| Pro | CCU(P) | 640 | 1.09 | Arg | CGU(R) | 388 | 0.74 |
| Pro | CCC(P) | 575 | 0.98 | Arg | CGC(R) | 242 | 0.46 |
| Pro | CCA(P) | 768 | 1.31 | Arg | CGA(R) | 576 | 1.09 |
| Pro | CCG(P) | 356 | 0.61 | Arg | CGG(R) | 333 | 0.63 |
| Thr | ACU(T) | 719 | 1.23 | Ser | AGU(S) | 707 | 0.89 |
| Thr | ACC(T) | 560 | 0.96 | Ser | AGC(S) | 462 | 0.58 |
| Thr | ACA(T) | 678 | 1.16 | Arg | AGA(R) | 1050 | 1.99 |
| Thr | ACG(T) | 379 | 0.65 | Arg | AGG(R) | 577 | 1.09 |
| Ala | GCU(A) | 474 | 1.29 | Gly | GGU(G) | 582 | 1.06 |
| Ala | GCC(A) | 351 | 0.95 | Gly | GGC(G) | 323 | 0.59 |
| Ala | GCA(A) | 418 | 1.14 | Gly | GGA(G) | 779 | 1.42 |
| Ala | GCG(A) | 230 | 0.62 | Gly | GGG(G) | 517 | 0.94 |
| *Ficus simplicissima* | | | | | | | |
| Phe | UUU(F) | 2351 | 1.24 | Tyr | UAU(Y) | 1715 | 1.41 |
| Phe | UUC(F) | 1450 | 0.76 | Tyr | UAC(Y) | 719 | 0.59 |
| Leu | UUA(L) | 1260 | 1.42 | Stop | UAA(*) | 1375 | 1.35 |
| Leu | UUG(L) | 1089 | 1.23 | Stop | UAG(*) | 766 | 0.75 |
| Leu | CUU(L) | 1132 | 1.27 | His | CAU(H) | 971 | 1.44 |
| Leu | CUC(L) | 612 | 0.69 | His | CAC(H) | 376 | 0.56 |
| Leu | CUA(L) | 758 | 0.85 | Gln | CAA(Q) | 1012 | 1.39 |
| Leu | CUG(L) | 482 | 0.54 | Gln | CAG(Q) | 446 | 0.61 |
| Ile | AUU(I) | 1971 | 1.23 | Asn | AAU(N) | 1995 | 1.43 |
| Ile | AUC(I) | 1092 | 0.68 | Asn | AAC(N) | 805 | 0.57 |
| Ile | AUA(I) | 1754 | 1.09 | Lys | AAA(K) | 2319 | 1.38 |
| Met | AUG(M) | 818 | 1 | Lys | AAG(K) | 1043 | 0.62 |
| Val | GUU(V) | 830 | 1.43 | Asp | GAU(D) | 1146 | 1.47 |
| Val | GUC(V) | 399 | 0.69 | Asp | GAC(D) | 417 | 0.53 |
| Val | GUA(V) | 697 | 1.2 | Glu | GAA(E) | 1391 | 1.4 |
| Val | GUG(V) | 393 | 0.68 | Glu | GAG(E) | 599 | 0.6 |
| Ser | UCU(S) | 1244 | 1.55 | Cys | UGU(C) | 763 | 1.27 |
| Ser | UCC(S) | 850 | 1.06 | Cys | UGC(C) | 435 | 0.73 |
| Ser | UCA(S) | 939 | 1.17 | Stop | UGA(*) | 923 | 0.9 |
| Ser | UCG(S) | 568 | 0.71 | Trp | UGG(W) | 659 | 1 |
| Pro | CCU(P) | 704 | 1.19 | Arg | CGU(R) | 383 | 0.72 |
| Pro | CCC(P) | 535 | 0.9 | Arg | CGC(R) | 250 | 0.47 |
| Pro | CCA(P) | 770 | 1.3 | Arg | CGA(R) | 590 | 1.11 |
| Pro | CCG(P) | 365 | 0.61 | Arg | CGG(R) | 360 | 0.68 |
| Thr | ACU(T) | 722 | 1.23 | Ser | AGU(S) | 750 | 0.93 |
| Thr | ACC(T) | 583 | 0.99 | Ser | AGC(S) | 470 | 0.58 |
| Thr | ACA(T) | 676 | 1.15 | Arg | AGA(R) | 1033 | 1.94 |
| Thr | ACG(T) | 363 | 0.62 | Arg | AGG(R) | 584 | 1.1 |
| Ala | GCU(A) | 540 | 1.41 | Gly | GGU(G) | 579 | 1.04 |
| Ala | GCC(A) | 346 | 0.91 | Gly | GGC(G) | 347 | 0.62 |
| Ala | GCA(A) | 428 | 1.12 | Gly | GGA(G) | 780 | 1.4 |
| Ala | GCG(A) | 213 | 0.56 | Gly | GGG(G) | 523 | 0.94 |
| *Ficus tinctoria* | | | | | | | |
| Phe | UUU(F) | 2425 | 1.27 | Tyr | UAU(Y) | 1649 | 1.4 |
| Phe | UUC(F) | 1403 | 0.73 | Tyr | UAC(Y) | 715 | 0.6 |
| Leu | UUA(L) | 1346 | 1.49 | Stop | UAA(*) | 1413 | 1.27 |
| Leu | UUG(L) | 1036 | 1.15 | Stop | UAG(*) | 767 | 0.69 |
| Leu | CUU(L) | 1073 | 1.19 | His | CAU(H) | 950 | 1.46 |
| Leu | CUC(L) | 653 | 0.73 | His | CAC(H) | 352 | 0.54 |
| Leu | CUA(L) | 835 | 0.93 | Gln | CAA(Q) | 1035 | 1.42 |
| Leu | CUG(L) | 460 | 0.51 | Gln | CAG(Q) | 420 | 0.58 |
| Ile | AUU(I) | 1892 | 1.21 | Asn | AAU(N) | 1956 | 1.43 |
| Ile | AUC(I) | 1065 | 0.68 | Asn | AAC(N) | 777 | 0.57 |
| Ile | AUA(I) | 1726 | 1.11 | Lys | AAA(K) | 2255 | 1.37 |
| Met | AUG(M) | 840 | 1 | Lys | AAG(K) | 1043 | 0.63 |
| Val | GUU(V) | 827 | 1.38 | Asp | GAU(D) | 1071 | 1.44 |
| Val | GUC(V) | 428 | 0.71 | Asp | GAC(D) | 417 | 0.56 |
| Val | GUA(V) | 743 | 1.24 | Glu | GAA(E) | 1302 | 1.38 |
| Val | GUG(V) | 397 | 0.66 | Glu | GAG(E) | 585 | 0.62 |
| Ser | UCU(S) | 1284 | 1.56 | Cys | UGU(C) | 706 | 1.24 |
| Ser | UCC(S) | 860 | 1.04 | Cys | UGC(C) | 435 | 0.76 |
| Ser | UCA(S) | 1035 | 1.26 | Stop | UGA(*) | 1162 | 1.04 |
| Ser | UCG(S) | 605 | 0.73 | Trp | UGG(W) | 747 | 1 |
| Pro | CCU(P) | 642 | 1.09 | Arg | CGU(R) | 395 | 0.72 |
| Pro | CCC(P) | 556 | 0.94 | Arg | CGC(R) | 227 | 0.41 |
| Pro | CCA(P) | 765 | 1.3 | Arg | CGA(R) | 582 | 1.06 |
| Pro | CCG(P) | 395 | 0.67 | Arg | CGG(R) | 359 | 0.65 |
| Thr | ACU(T) | 683 | 1.17 | Ser | AGU(S) | 683 | 0.83 |
| Thr | ACC(T) | 592 | 1.01 | Ser | AGC(S) | 481 | 0.58 |
| Thr | ACA(T) | 713 | 1.22 | Arg | AGA(R) | 1136 | 2.07 |
| Thr | ACG(T) | 352 | 0.6 | Arg | AGG(R) | 592 | 1.08 |
| Ala | GCU(A) | 456 | 1.23 | Gly | GGU(G) | 548 | 1.03 |
| Ala | GCC(A) | 342 | 0.92 | Gly | GGC(G) | 323 | 0.61 |
| Ala | GCA(A) | 452 | 1.22 | Gly | GGA(G) | 763 | 1.43 |
| Ala | GCG(A) | 230 | 0.62 | Gly | GGG(G) | 498 | 0.93 |
