# Supplementary material for: Comprehensive analysis of complete chloroplast genome and phylogenetic aspects of ten Ficus species
Source: BMC Plant Biol. 2022 May 23;22:253. doi: 10.1186/s12870-022-03643-4 (PMC9125854; doi:10.1186/s12870-022-03643-4)
Supplement: Supplementary file 6 — Additional file 6: Table S5. Predicted RNA editing sites in eleven Ficus chloroplast genomes by the PREP program. [file 12870_2022_3643_MOESM6_ESM.doc]

**Table S5.** Predicted RNA editing sites in eleven *Ficus* chloroplast genomes by the PREP program

|  |  | *Ficus pumila* |  |  |
| --- | --- | --- | --- | --- |
| Gene | Nucleotide position | Codon change | Amino acid change | Score |
| accD | 13 | CGG-TGG | R-E | 1.00 |
|  | 344 | CCA-CTA | P-L | 1.00 |
|  | 1421 | CCT-CTT | P-L | 1.00 |
| atpA | 773 | TCA-TTA | S-L | 1.00 |
|  | 791 | CCT-CTT | P-L | 1.00 |
|  | 914 | TCA-TTA | S-L | 1.00 |
| atpB | 37 | CCC-TCC | P-S | 1.00 |
| atpF | 19 | CCT-TCT | P-S | 0.86 |
|  | 92 | CCA-CTA | P-L | 0.86 |
| atpI | 76 | CTC-TTC | L-F | 0.86 |
| clpP | 559 | CAC-TAC | H-Y | 1.00 |
| matK | 52 | CTC-TTC | L-F | 1.00 |
|  | 640 | CAT-TAT | H-Y | 1.00 |
|  | 1187 | TCA-TTA | S-L | 0.86 |
| ndhA | 107 | CCT-CTT | P-L | 1.00 |
|  | 341 | TCA-TTA | S-L | 1.00 |
|  | 566 | TCA-TTA | S-L | 1.00 |
|  | 1073 | TCC-TTC | S-F | 1.00 |
| ndhB | 1682 | TCA-TTA | S-L | 1.00 |
|  | 2000 | CCA-CTA | P-L | 1.00 |
|  | 2075 | ACG-ATG | T-M | 1.00 |
|  | 2119 | CAT-TAT | H-Y | 1.00 |
|  | 2144 | TCA-TTA | S-L | 0.80 |
|  | 2270 | CCA-CTA | P-L | 1.00 |
|  | 2279 | TCT-TTT | S-F | 1.00 |
|  | 2363 | TCA-TTA | S-L | 1.00 |
|  | 2369 | TCA-TTA | S-L | 1.00 |
|  | 2645 | TCA-TTA | S-L | 1.00 |
|  | 2788 | CAT-TAT | H-Y | 1.00 |
|  | 3014 | CCA-CTA | P-L | 1.00 |
| ndhD | 29 | ACG-ATG | T-M | 1.00 |
|  | 410 | CCA-CTA | P-L | 1.00 |
|  | 626 | TCA-TTA | S-L | 1.00 |
|  | 701 | TCG-TTG | S-L | 1.00 |
|  | 905 | TCA-TTA | S-L | 11.00 |
|  | 914 | CCT-CTT | P-L | 1.00 |
|  | 1103 | GCT-GTT | A-V | 1.00 |
| ndhF | 196 | CCT-TCT | P-S | 1.00 |
|  | 1867 | CTT-TTT | L-F | 1.00 |
|  | 1991 | ACA-ATA | T-I | 0.80 |
| ndhG | 314 | ACA-ATA | T-I | 0.80 |
|  | 494 | GCG-GTG | A-V | 0.80 |
| psaI | 83 | TCT-TTT | S-F | 0.86 |
| psbE | 214 | CCT-TCT | P-S | 1.00 |
| psbF | 77 | TCT-TTT | S-F | 1.00 |
| psbL | 2 | ACG-ATG | T-M | 1.00 |
| rpl2 | 1372 | CCC-TCC | P-S | 1.00 |
| rpoA | 821 | TCA-TTA | S-L | 1.00 |
| rpoB | 338 | TCT-TTT | S-F | 1.00 |
|  | 2426 | TCA-TTA | S-L | 0.86 |
| rpoC1 | 41 | TCA-TTA | S-L | 1.00 |
|  | 1562 | ACG-ATG | T-M | 0.86 |
| rpoC2 | 1493 | ACG-ATG | T-M | 0.86 |
|  | 1600 | CTT-TTT | L-F | 1.00 |
|  | 1795 | CGT-TGT | R-G | 0.86 |
|  | 1975 | CTT-TTT | S-L | 0.83 |
| rps2 | 248 | TCA-TTA | S-L | 1.00 |
| rps14 | 80 | TCA-TTA | S-L | 1.00 |
| rps16 | 212 | TCA-TTA | S-L | 0.83 |
| *Ficus tikoua* | | | | |
| accD | 13 | CGG-TGG | R-W | 1.00 |
|  | 344 | CCA-CTA | P-L | 1.00 |
|  | 1421 | CCT-CTT | P-L | 1.00 |
| atpA | 773 | TCA-TTA | S-L | 1.00 |
|  | 791 | CCT-CTT | P-L | 1.00 |
|  | 914 | TCA-TTA | S-L | 1.00 |
| atpB | 37 | CCC-TCC | P-S | 1.00 |
| atpF | 19 | CCT-TCT | P-S | 0.86 |
|  | 92 | CCA-CTA | P-L | 0.86 |
| atpI | 76 | CTC-TTC | L-F | 0.86 |
| clpP | 559 | CAC-TAC | H-Y | 1.00 |
| matK | 52 | CTC-TTC | L-F | 1.00 |
|  | 640 | CAT-TAT | H-Y | 1.00 |
|  | 1187 | TCA-TTA | S-L | 0.86 |
| ndhA | 107 | CCT-CTT | P-L | 1.00 |
|  | 341 | TCA-TTA | S-L | 1.00 |
|  | 566 | TCA-TTA | S-L | 1.00 |
|  | 1073 | TCC-TTC | S-F | 1.00 |
| ndhB | 1682 | TCA-TTA | S-L | 1.00 |
|  | 2000 | CCA-CTA | P-L | 1.00 |
|  | 2075 | ACG-ATG | T-M | 1.00 |
|  | 2119 | CAT-TAT | H-Y | 1.00 |
|  | 2144 | TCA-TTA | S-L | 0.80 |
|  | 2270 | CCA-CTA | P-L | 1.00 |
|  | 2279 | TCT-TTT | S-F | 1.00 |
|  | 2363 | TCA-TTA | S-L | 1.00 |
|  | 2369 | TCA-TTA | S-L | 1.00 |
|  | 2645 | TCA-TTA | S-L | 1.00 |
|  | 2788 | CAT-TAT | H-Y | 1.00 |
|  | 3014 | CCA-CTA | P-L | 1.00 |
| ndhD | 29 | ACG-ATG | T-M | 1.00 |
|  | 340 | CGG-TGG | R-W | 0.80 |
|  | 410 | CCA-CTA | P-L | 1.00 |
|  | 626 | TCA-TTA | S-L | 1.00 |
|  | 701 | TCG-TTG | S-L | 1.00 |
|  | 905 | TCA-TTA | S-L | 11.00 |
|  | 914 | CCT-CTT | P-L | 1.00 |
|  | 1103 | GCT-GTT | A-V | 1.00 |
|  | 1325 | TCA-TTA | S-L | 0.80 |
|  | 1432 | CTT-TTT | L-F | 0.80 |
| ndhF | 196 | CCT-TCT | P-S | 1.00 |
|  | 905 | ACT-ATT | T-I | 1.00 |
|  | 1711 | CAT-TAT | H-Y | 1.00 |
|  | 2201 | TCG-TTG | S-L | 1.00 |
|  | 2948 | TCT-TTT | S-F | 1.00 |
| ndhG | 314 | ACA-ATA | T-I | 0.80 |
|  | 494 | GCG-GTG | A-V | 0.80 |
| psaI | 83 | TCT-TTT | S-F | 0.86 |
| psbE | 214 | CCT-TCT | P-S | 1.00 |
| psbF | 77 | TCT-TTT | S-F | 1.00 |
| psbL | 2 | ACG-ATG | T-M | 1.00 |
| rpl2 | 1372 | CCC-TCC | P-S | 1.00 |
| rpoA | 821 | TCA-TTA | S-L | 1.00 |
| rpoB | 338 | TCT-TTT | S-F | 1.00 |
|  | 2426 | TCA-TTA | S-L | 0.86 |
| rpoC1 | 41 | TCA-TTA | S-L | 1.00 |
|  | 1562 | ACG-ATG | T-M | 0.86 |
| rpoC2 | 1493 | ACG-ATG | T-M | 0.86 |
|  | 1600 | CTT-TTT | L-F | 1.00 |
|  | 1795 | CGT-TGT | R-G | 0.86 |
|  | 1975 | CTT-TTT | S-L | 0.83 |
| rps2 | 248 | TCA-TTA | S-L | 1.00 |
| rps14 | 80 | TCA-TTA | S-L | 1.00 |
| rps16 | 212 | TCA-TTA | S-L | 0.83 |
| *Ficus hispida* | | | | |
| accD | 13 | CGG-TGG | R-W | 1.00 |
|  | 344 | CCA-CTA | P-L | 1.00 |
|  | 734 | TCT-TTT | S-F | 1.00 |
|  | 1421 | CCT-CTT | P-L | 1.00 |
| atpA | 773 | TCA-TTA | S-L | 1.00 |
|  | 791 | CCT-CTT | P-L | 1.00 |
|  | 914 | TCA-TTA | S-L | 1.00 |
| atpB | 37 | CCC-TCC | P-S | 1.00 |
| atpF | 19 | CCT-TCT | P-S | 0.86 |
|  | 92 | CCA-CTA | P-L | 0.86 |
| atpI | 76 | CTC-TTC | L-F | 0.86 |
| clpP | 559 | CAC-TAC | H-Y | 1.00 |
| matK | 52 | CTC-TTC | L-F | 1.00 |
|  | 646 | CAT-TAT | H-Y | 1.00 |
|  | 1193 | TCA-TTA | S-L | 0.86 |
| ndhA | 107 | CCT-CTT | P-L | 1.00 |
|  | 341 | TCA-TTA | S-L | 1.00 |
|  | 566 | TCA-TTA | S-L | 1.00 |
|  | 1073 | TCC-TTC | S-F | 1.00 |
| ndhB | 1682 | TCA-TTA | S-L | 1.00 |
|  | 2000 | CCA-CTA | P-L | 1.00 |
|  | 2075 | ACG-ATG | T-M | 1.00 |
|  | 2119 | CAT-TAT | H-Y | 1.00 |
|  | 2144 | TCA-TTA | S-L | 0.80 |
|  | 2270 | CCA-CTA | P-L | 1.00 |
|  | 2279 | TCT-TTT | S-F | 1.00 |
|  | 2363 | TCA-TTA | S-L | 1.00 |
|  | 2369 | TCA-TTA | S-L | 1.00 |
|  | 2645 | TCA-TTA | S-L | 1.00 |
|  | 2788 | CAT-TAT | H-Y | 1.00 |
|  | 3014 | CCA-CTA | P-L | 1.00 |
| ndhD | 29 | ACG-ATG | T-M | 1.00 |
|  | 340 | CGG-TGG | R-W | 0.80 |
|  | 410 | CCA-CTA | P-L | 1.00 |
|  | 626 | TCA-TTA | S-L | 1.00 |
|  | 701 | TCG-TTG | S-L | 1.00 |
|  | 905 | TCA-TTA | S-L | 11.00 |
|  | 914 | CCT-CTT | P-L | 1.00 |
|  | 1103 | GCT-GTT | A-V | 1.00 |
|  | 1325 | TCA-TTA | S-L | 0.80 |
|  | 1432 | CTT-TTT | L-F | 0.80 |
| ndhF | 196 | CCT-TCT | P-S | 1.00 |
|  | 1876 | CTT-TTT | L-F | 1.00 |
|  | 2000 | ACA-ATA | T-I | 0.80 |
| ndhG | 314 | ACA-ATA | T-I | 0.80 |
|  | 494 | GCG-GTG | A-V | 0.80 |
| psaI | 83 | TCT-TTT | S-F | 0.86 |
| psbE | 214 | CCT-TCT | P-S | 1.00 |
| psbF | 77 | TCT-TTT | S-F | 1.00 |
| psbL | 2 | ACG-ATG | T-M | 1.00 |
| rpl2 | 1372 | CCC-TCC | P-S | 1.00 |
| rpoA | 821 | TCA-TTA | S-L | 1.00 |
| rpoB | 338 | TCT-TTT | S-F | 1.00 |
|  | 2426 | TCA-TTA | S-L | 0.86 |
| rpoC1 | 41 | TCA-TTA | S-L | 1.00 |
|  | 1562 | ACG-ATG | T-M | 0.86 |
| rpoC2 | 1493 | ACG-ATG | T-M | 0.86 |
|  | 1594 | CTT-TTT | L-F | 1.00 |
|  | 1789 | CGT-TGT | R-G | 0.86 |
|  | 1969 | CTT-TTT | S-L | 0.83 |
| rps2 | 248 | TCA-TTA | S-L | 1.00 |
| rps14 | 80 | TCA-TTA | S-L | 1.00 |
| rps16 | 212 | TCA-TTA | S-L | 0.83 |
| *Ficus virens* | | | | |
| accD | 13 | CGG-TGG | R-W | 1.00 |
|  | 344 | CCA-CTA | P-L | 1.00 |
|  | 1421 | CCT-CTT | P-L | 1.00 |
| atpA | 773 | TCA-TTA | S-L | 1.00 |
|  | 791 | CCT-CTT | P-L | 1.00 |
|  | 914 | TCA-TTA | S-L | 1.00 |
| atpB | 37 | CCC-TCC | P-S | 1.00 |
| atpF | 19 | CCT-TCT | P-S | 0.86 |
|  | 92 | CCA-CTA | P-L | 0.86 |
| atpI | 76 | CTC-TTC | L-F | 0.86 |
| clpP | 559 | CAC-TAC | H-Y | 1.00 |
| matK | 52 | CTC-TTC | L-F | 1.00 |
|  | 640 | CAT-TAT | H-Y | 1.00 |
|  | 1187 | TCA-TTA | S-L | 0.86 |
| ndhA | 107 | CCT-CTT | P-L | 1.00 |
|  | 341 | TCA-TTA | S-L | 1.00 |
|  | 566 | TCA-TTA | S-L | 1.00 |
|  | 1073 | TCC-TTC | S-F | 1.00 |
| ndhB | 1682 | TCA-TTA | S-L | 1.00 |
|  | 2000 | CCA-CTA | P-L | 1.00 |
|  | 2075 | ACG-ATG | T-M | 1.00 |
|  | 2119 | CAT-TAT | H-Y | 1.00 |
|  | 2144 | TCA-TTA | S-L | 0.80 |
|  | 2270 | CCA-CTA | P-L | 1.00 |
|  | 2279 | TCT-TTT | S-F | 1.00 |
|  | 2363 | TCA-TTA | S-L | 1.00 |
|  | 2369 | TCA-TTA | S-L | 1.00 |
|  | 2645 | TCA-TTA | S-L | 1.00 |
|  | 2788 | CAT-TAT | H-Y | 1.00 |
|  | 3014 | CCA-CTA | P-L | 1.00 |
| ndhD | 29 | ACG-ATG | T-M | 1.00 |
|  | 340 | CGG-TGG | R-W | 0.80 |
|  | 410 | CCA-CTA | P-L | 1.00 |
|  | 626 | TCA-TTA | S-L | 1.00 |
|  | 701 | TCG-TTG | S-L | 1.00 |
|  | 905 | TCA-TTA | S-L | 11.00 |
|  | 914 | CCT-CTT | P-L | 1.00 |
|  | 1103 | GCT-GTT | A-V | 1.00 |
|  | 1325 | TCA-TTA | S-L | 0.80 |
|  | 1432 | CTT-TTT | L-F | 0.80 |
| ndhF | 196 | CCT-TCT | P-S | 1.00 |
|  | 1876 | CTT-TTT | L-F | 1.00 |
|  | 2000 | ACA-ATA | T-I | 0.80 |
| ndhG | 314 | ACA-ATA | T-I | 0.80 |
|  | 494 | GCG-GTG | A-V | 0.80 |
| psaI | 83 | TCT-TTT | S-F | 0.86 |
| psbE | 214 | CCT-TCT | P-S | 1.00 |
| psbF | 77 | TCT-TTT | S-F | 1.00 |
| psbL | 2 | ACG-ATG | T-W | 1.00 |
| rpl2 | 1372 | CCC-TCC | P-S | 1.00 |
| rpoA | 821 | TCA-TTA | S-L | 1.00 |
| rpoB | 338 | TCT-TTT | S-F | 1.00 |
|  | 2426 | TCA-TTA | S-L | 0.86 |
| rpoC1 | 41 | TCA-TTA | S-L | 1.00 |
|  | 1562 | ACG-ATG | T-M | 0.86 |
| rpoC2 | 1505 | ACG-ATG | T-M | 0.86 |
|  | 1612 | CTT-TTT | L-F | 1.00 |
|  | 1807 | CGT-TGT | R-G | 0.86 |
|  | 1987 | CTT-TTT | S-L | 0.83 |
| rps2 | 248 | TCA-TTA | S-L | 1.00 |
| rps14 | 80 | TCA-TTA | S-L | 1.00 |
| rps16 | 212 | TCA-TTA | S-L | 0.83 |
| *Ficus sarmentosa var.impressa* | | | | |
| accD | 344 | CCA-CTA | P-L | 1.00 |
|  | 1421 | CCT-CTT | P-L | 1.00 |
| atpA | 773 | TCA-TTA | S-L | 1.00 |
|  | 791 | CCT-CTT | P-L | 1.00 |
|  | 914 | TCA-TTA | S-L | 1.00 |
| atpB | 37 | CCC-TCC | P-S | 1.00 |
| atpF | 19 | CCT-TCT | P-S | 0.86 |
|  | 92 | CCA-CTA | P-L | 0.86 |
| atpI | 76 | CTC-TTC | L-F | 0.86 |
| clpP | 559 | CAC-TAC | H-Y | 1.00 |
| matK | 52 | CTC-TTC | L-F | 1.00 |
|  | 646 | CAT-TAT | H-Y | 1.00 |
|  | 1193 | TCA-TTA | S-L | 0.86 |
| ndhA | 107 | CCT-CTT | P-L | 1.00 |
|  | 341 | TCA-TTA | S-L | 1.00 |
|  | 566 | TCA-TTA | S-L | 1.00 |
|  | 1073 | TCC-TTC | S-F | 1.00 |
| ndhB | 1682 | TCA-TTA | S-L | 1.00 |
|  | 2000 | CCA-CTA | P-L | 1.00 |
|  | 2075 | ACG-ATG | T-M | 1.00 |
|  | 2119 | CAT-TAT | H-Y | 1.00 |
|  | 2144 | TCA-TTA | S-L | 0.80 |
|  | 2270 | CCA-CTA | P-L | 1.00 |
|  | 2279 | TCT-TTT | S-F | 1.00 |
|  | 2363 | TCA-TTA | S-L | 1.00 |
|  | 2369 | TCA-TTA | S-L | 1.00 |
|  | 2645 | TCA-TTA | S-L | 1.00 |
|  | 2788 | CAT-TAT | H-Y | 1.00 |
|  | 3014 | CCA-CTA | P-L | 1.00 |
| ndhD | 29 | ACG-ATG | T-M | 1.00 |
|  | 340 | CGG-TGG | R-W | 0.80 |
|  | 410 | CCA-CTA | P-L | 1.00 |
|  | 626 | TCA-TTA | S-L | 1.00 |
|  | 701 | TCG-TTG | S-L | 1.00 |
|  | 905 | TCA-TTA | S-L | 11.00 |
|  | 914 | CCT-CTT | P-L | 1.00 |
|  | 1103 | GCT-GTT | A-V | 1.00 |
|  | 1325 | TCA-TTA | S-L | 0.80 |
|  | 1432 | CTT-TTT | L-F | 0.80 |
| ndhF | 196 | CCT-TCT | P-S | 1.00 |
|  | 1876 | CTT-TTT | L-F | 1.00 |
|  | 2000 | ACA-ATA | T-I | 0.80 |
|  | 2246 | TCT-TTT | S-F | 1.00 |
|  | 2257 | CTT-TTT | L-F | 1.00 |
| ndhG | 314 | ACA-ATA | T-I | 0.80 |
|  | 494 | GCG-GTG | A-V | 0.80 |
| psaI | 83 | TCT-TTT | S-F | 0.86 |
| psbE | 214 | CCT-TCT | P-S | 1.00 |
| psbF | 77 | TCT-TTT | S-F | 1.00 |
| psbL | 2 | ACG-ATG | T-M | 1.00 |
| rpl2 | 1372 | CCC-TCC | P-S | 1.00 |
| rpoA | 821 | TCA-TTA | S-L | 1.00 |
| rpoB | 338 | TCT-TTT | S-F | 1.00 |
|  | 2426 | TCA-TTA | S-L | 0.86 |
| rpoC1 | 41 | TCA-TTA | S-L | 1.00 |
|  | 1562 | ACG-ATG | T-M | 0.86 |
| rpoC2 | 1505 | ACG-ATG | T-M | 0.86 |
|  | 1612 | CTT-TTT | L-F | 1.00 |
|  | 1807 | CGT-TGT | R-G | 0.86 |
|  | 1987 | CTT-TTT | S-L | 0.83 |
| rps2 | 248 | TCA-TTA | S-L | 1.00 |
| rps14 | 80 | TCA-TTA | S-L | 1.00 |
| rps16 | 212 | TCA-TTA | S-L | 0.83 |
| *Ficus pandurata* | | | | |
| accD | 13 | CGG-TGG | R-W | 1.00 |
|  | 344 | CCA-CTA | P-L | 1.00 |
|  | 734 | TCT-TTT | S-F | 1.00 |
|  | 1421 | CCT-CTT | P-L | 1.00 |
| atpA | 773 | TCA-TTA | S-L | 1.00 |
|  | 791 | CCT-CTT | P-L | 1.00 |
|  | 914 | TCA-TTA | S-L | 1.00 |
| atpB | 37 | CCC-TCC | P-S | 1.00 |
| atpF | 19 | CCT-TCT | P-S | 0.86 |
|  | 92 | CCA-CTA | P-L | 0.86 |
| atpI | 76 | CTC-TTC | L-F | 0.86 |
| clpP | 530 | GCT-GTT | A-V | 1.00 |
|  | 559 | CAC-TAC | H-Y | 1.00 |
| matK | 640 | CAT-TAT | H-Y | 1.00 |
|  | 1187 | TCA-TTA | S-L | 0.86 |
| ndhA | 107 | CCT-CTT | P-L | 1.00 |
|  | 341 | TCA-TTA | S-L | 1.00 |
|  | 566 | TCA-TTA | S-L | 1.00 |
|  | 1073 | TCC-TTC | S-F | 1.00 |
| ndhB | 1682 | TCA-TTA | S-L | 1.00 |
|  | 2000 | CCA-CTA | P-L | 1.00 |
|  | 2075 | ACG-ATG | T-M | 1.00 |
|  | 2119 | CAT-TAT | H-Y | 1.00 |
|  | 2144 | TCA-TTA | S-L | 0.80 |
|  | 2270 | CCA-CTA | P-L | 1.00 |
|  | 2279 | TCT-TTT | S-F | 1.00 |
|  | 2363 | TCA-TTA | S-L | 1.00 |
|  | 2369 | TCA-TTA | S-L | 1.00 |
|  | 2645 | TCA-TTA | S-L | 1.00 |
|  | 2788 | CAT-TAT | H-Y | 1.00 |
|  | 3014 | CCA-CTA | P-L | 1.00 |
| ndhD | 29 | ACG-ATG | T-M | 1.00 |
|  | 340 | CGG-TGG | R-W | 0.80 |
|  | 410 | CCA-CTA | P-L | 1.00 |
|  | 626 | TCA-TTA | S-L | 1.00 |
|  | 701 | TCG-TTG | S-L | 1.00 |
|  | 905 | TCA-TTA | S-L | 11.00 |
|  | 914 | CCT-CTT | P-L | 1.00 |
|  | 1103 | GCT-GTT | A-V | 1.00 |
|  | 1325 | TCA-TTA | S-L | 0.80 |
|  | 1432 | CTT-TTT | L-F | 0.80 |
| ndhF | 196 | CCT-TCT | P-S | 1.00 |
|  | 1876 | CTT-TTT | L-F | 1.00 |
|  | 2000 | ACA-ATA | T-I | 0.80 |
| ndhG | 314 | ACA-ATA | T-I | 0.80 |
|  | 494 | GCG-GTG | A-V | 0.80 |
| psaI | 83 | TCT-TTT | S-F | 0.86 |
| psbE | 214 | CCT-TCT | P-S | 1.00 |
| psbF | 77 | TCT-TTT | S-F | 1.00 |
| psbL | 2 | ACG-ATG | T-M | 1.00 |
| rpl2 | 1372 | CCC-TCC | P-S | 1.00 |
| rpoA | 821 | TCA-TTA | S-L | 1.00 |
| rpoB | 338 | TCT-TTT | S-F | 1.00 |
|  | 2426 | TCA-TTA | S-L | 0.86 |
| rpoC1 | 41 | TCA-TTA | S-L | 1.00 |
|  | 1562 | ACG-ATG | T-M | 0.86 |
| rpoC2 | 1493 | ACG-ATG | T-M | 0.86 |
|  | 1600 | CTT-TTT | L-F | 1.00 |
|  | 1795 | CGT-TGT | R-G | 0.86 |
|  | 1975 | CTT-TTT | S-L | 0.83 |
| rps2 | 248 | TCA-TTA | S-L | 1.00 |
| rps14 | 80 | TCA-TTA | S-L | 1.00 |
| rps16 | 212 | TCA-TTA | S-L | 0.83 |
| *Ficus microcarpa* | | | | |
| accD | 13 | CGG-TGG | R-W | 1.00 |
|  | 344 | CCA-CTA | P-L | 1.00 |
|  | 734 | TCT-TTT | S-F | 1.00 |
|  | 1421 | CCT-CTT | P-L | 1.00 |
| atpA | 773 | TCA-TTA | S-L | 1.00 |
|  | 791 | CCT-CTT | P-L | 1.00 |
|  | 914 | TCA-TTA | S-L | 1.00 |
| atpB | 37 | CCC-TCC | P-S | 1.00 |
| atpF | 19 | CCT-TCT | P-S | 0.86 |
|  | 92 | CCA-CTA | P-L | 0.86 |
| atpI | 76 | CTC-TTC | L-F | 0.86 |
| clpP | 559 | CAC-TAC | H-Y | 1.00 |
| matK | 52 | CTC-TTC | L-F | 1.00 |
|  | 640 | CAT-TAT | H-Y | 1.00 |
|  | 1187 | TCA-TTA | S-L | 0.86 |
| ndhA | 107 | CCT-CTT | P-L | 1.00 |
|  | 341 | TCA-TTA | S-L | 1.00 |
|  | 566 | TCA-TTA | S-L | 1.00 |
|  | 1073 | TCC-TTC | S-F | 1.00 |
| ndhB | 1682 | TCA-TTA | S-L | 1.00 |
|  | 2000 | CCA-CTA | P-L | 1.00 |
|  | 2075 | ACG-ATG | T-M | 1.00 |
|  | 2119 | CAT-TAT | H-Y | 1.00 |
|  | 2144 | TCA-TTA | S-L | 0.80 |
|  | 2270 | CCA-CTA | P-L | 1.00 |
|  | 2279 | TCT-TTT | S-F | 1.00 |
|  | 2363 | TCA-TTA | S-L | 1.00 |
|  | 2369 | TCA-TTA | S-L | 1.00 |
|  | 2645 | TCA-TTA | S-L | 1.00 |
|  | 2788 | CAT-TAT | H-Y | 1.00 |
|  | 3014 | CCA-CTA | P-L | 1.00 |
| ndhD | 29 | ACG-ATG | T-M | 1.00 |
|  | 340 | CGG-TGG | R-W | 0.80 |
|  | 410 | CCA-CTA | P-L | 1.00 |
|  | 626 | TCA-TTA | S-L | 1.00 |
|  | 701 | TCG-TTG | S-L | 1.00 |
|  | 905 | TCA-TTA | S-L | 11.00 |
|  | 914 | CCT-CTT | P-L | 1.00 |
|  | 1103 | GCT-GTT | A-V | 1.00 |
|  | 1325 | TCA-TTA | S-L | 0.80 |
|  | 1432 | CTT-TTT | L-F | 0.80 |
| ndhF | 196 | CCT-TCT | P-S | 1.00 |
|  | 1876 | CTT-TTT | L-F | 1.00 |
|  | 2000 | ACA-ATA | T-I | 0.80 |
| ndhG | 314 | ACA-ATA | T-I | 0.80 |
|  | 494 | GCG-GTG | A-V | 0.80 |
| psaI | 83 | TCT-TTT | S-F | 0.86 |
| psbE | 214 | CCT-TCT | P-S | 1.00 |
| psbF | 77 | TCT-TTT | S-F | 1.00 |
| psbL | 2 | ACG-ATG | T-M | 1.00 |
| rpl2 | 1372 | CCC-TCC | P-S | 1.00 |
| rpoA | 821 | TCA-TTA | S-L | 1.00 |
| rpoB | 338 | TCT-TTT | S-F | 1.00 |
|  | 2426 | TCA-TTA | S-L | 0.86 |
| rpoC1 | 41 | TCA-TTA | S-L | 1.00 |
|  | 1562 | ACG-ATG | T-M | 0.86 |
| rpoC2 | 1505 | ACG-ATG | T-M | 0.86 |
|  | 1612 | CTT-TTT | L-F | 1.00 |
|  | 1807 | CGT-TGT | R-G | 0.86 |
|  | 1987 | CTT-TTT | S-L | 0.83 |
| rps2 | 248 | TCA-TTA | S-L | 1.00 |
| rps14 | 80 | TCA-TTA | S-L | 1.00 |
| rps16 | 212 | TCA-TTA | S-L | 0.83 |
| *Ficus formosana* | | | | |
| accD | 13 | CGG-TGG | R-W | 1.00 |
|  | 344 | CCA-CTA | P-L | 1.00 |
|  | 734 | TCT-TTT | S-F | 1.00 |
|  | 1421 | CCT-CTT | P-L | 1.00 |
| atpA | 773 | TCA-TTA | S-L | 1.00 |
|  | 791 | CCT-CTT | P-L | 1.00 |
|  | 914 | TCA-TTA | S-L | 1.00 |
| atpB | 37 | CCC-TCC | P-S | 1.00 |
| atpF | 19 | CCT-TCT | P-S | 0.86 |
|  | 92 | CCA-CTA | P-L | 0.86 |
| atpI | 76 | CTC-TTC | L-F | 0.86 |
| clpP | 559 | CAC-TAC | H-Y | 1.00 |
| matK | 52 | CTC-TTC | L-F | 1.00 |
|  | 640 | CAT-TAT | H-Y | 1.00 |
|  | 1187 | TCA-TTA | S-L | 0.86 |
| ndhA | 107 | CCT-CTT | P-L | 1.00 |
|  | 341 | TCA-TTA | S-L | 1.00 |
|  | 566 | TCA-TTA | S-L | 1.00 |
|  | 1073 | TCC-TTC | S-F | 1.00 |
| ndhB | 1682 | TCA-TTA | S-L | 1.00 |
|  | 2000 | CCA-CTA | P-L | 1.00 |
|  | 2075 | ACG-ATG | T-M | 1.00 |
|  | 2119 | CAT-TAT | H-Y | 1.00 |
|  | 2144 | TCA-TTA | S-L | 0.80 |
|  | 2270 | CCA-CTA | P-L | 1.00 |
|  | 2279 | TCT-TTT | S-F | 1.00 |
|  | 2363 | TCA-TTA | S-L | 1.00 |
|  | 2369 | TCA-TTA | S-L | 1.00 |
|  | 2645 | TCA-TTA | S-L | 1.00 |
|  | 2788 | CAT-TAT | H-Y | 1.00 |
|  | 3014 | CCA-CTA | P-L | 1.00 |
| ndhD | 329 | ACG-ATG | T-M | 1.00 |
|  | 640 | CGG-TGG | R-W | 0.80 |
|  | 710 | CCA-CTA | P-L | 1.00 |
|  | 926 | TCA-TTA | S-L | 1.00 |
|  | 1001 | TCG-TTG | S-L | 1.00 |
|  | 1205 | TCA-TTA | S-L | 11.00 |
|  | 1214 | CCT-CTT | P-L | 1.00 |
|  | 1403 | GCT-GTT | A-V | 1.00 |
|  | 1625 | TCA-TTA | S-L | 0.80 |
|  | 1732 | CTT-TTT | L-F | 0.80 |
| ndhF | 196 | CCT-TCT | P-S | 1.00 |
|  | 1876 | CTT-TTT | L-F | 1.00 |
|  | 2000 | ACA-ATA | T-I | 0.80 |
| ndhG | 314 | ACA-ATA | T-I | 0.80 |
|  | 494 | GCG-GTG | A-V | 0.80 |
| psaI | 83 | TCT-TTT | S-F | 0.86 |
| psbE | 214 | CCT-TCT | P-S | 1.00 |
| psbF | 77 | TCT-TTT | S-F | 1.00 |
| psbL | 2 | ACG-ATG | T-M | 1.00 |
| rpl2 | 1372 | CCC-TCC | P-S | 1.00 |
| rpoA | 821 | TCA-TTA | S-L | 1.00 |
| rpoB | 338 | TCT-TTT | S-F | 1.00 |
|  | 2426 | TCA-TTA | S-L | 0.86 |
| rpoC1 | 41 | TCA-TTA | S-L | 1.00 |
|  | 1562 | ACG-ATG | T-M | 0.86 |
| rpoC2 | 1505 | ACG-ATG | T-M | 0.86 |
|  | 1612 | CTT-TTT | L-F | 1.00 |
|  | 1807 | CGT-TGT | R-G | 0.86 |
|  | 1987 | CTT-TTT | S-L | 0.83 |
| rps2 | 248 | TCA-TTA | S-L | 1.00 |
| rps14 | 80 | TCA-TTA | S-L | 1.00 |
| rps16 | 212 | TCA-TTA | S-L | 0.83 |
| *Ficus sarmentosa var. lacrymans* | | | | |
| accD | 13 | CGG-TGG | R-W | 1.00 |
|  | 344 | CCA-CTA | P-L | 1.00 |
|  | 734 | TCT-TTT | S-F | 1.00 |
|  | 1421 | CCT-CTT | P-L | 1.00 |
| atpA | 773 | TCA-TTA | S-L | 1.00 |
|  | 791 | CCT-CTT | P-L | 1.00 |
|  | 914 | TCA-TTA | S-L | 1.00 |
| atpB | 37 | CCC-TCC | P-S | 1.00 |
| atpF | 19 | CCT-TCT | P-S | 0.86 |
|  | 92 | CCA-CTA | P-L | 0.86 |
| atpI | 76 | CTC-TTC | L-F | 0.86 |
| clpP | 562 | CAC-TAC | H-Y | 1.00 |
| matK | 52 | CTC-TTC | L-F | 1.00 |
|  | 640 | CAT-TAT | H-Y | 1.00 |
|  | 1187 | TCA-TTA | S-L | 0.86 |
| ndhA | 107 | CCT-CTT | P-L | 1.00 |
|  | 341 | TCA-TTA | S-L | 1.00 |
|  | 566 | TCA-TTA | S-L | 1.00 |
|  | 1073 | TCC-TTC | S-F | 1.00 |
| ndhB | 1682 | TCA-TTA | S-L | 1.00 |
|  | 2000 | CCA-CTA | P-L | 1.00 |
|  | 2075 | ACG-ATG | T-M | 1.00 |
|  | 2119 | CAT-TAT | H-Y | 1.00 |
|  | 2144 | TCA-TTA | S-L | 0.80 |
|  | 2270 | CCA-CTA | P-L | 1.00 |
|  | 2279 | TCT-TTT | S-F | 1.00 |
|  | 2363 | TCA-TTA | S-L | 1.00 |
|  | 2369 | TCA-TTA | S-L | 1.00 |
|  | 2645 | TCA-TTA | S-L | 1.00 |
|  | 2788 | CAT-TAT | H-Y | 1.00 |
|  | 3014 | CCA-CTA | P-L | 1.00 |
| ndhD | 29 | ACG-ATG | T-M | 1.00 |
|  | 340 | CGG-TGG | R-W | 0.80 |
|  | 410 | CCA-CTA | P-L | 1.00 |
|  | 626 | TCA-TTA | S-L | 1.00 |
|  | 701 | TCG-TTG | S-L | 1.00 |
|  | 905 | TCA-TTA | S-L | 11.00 |
|  | 914 | CCT-CTT | P-L | 1.00 |
|  | 1103 | GCT-GTT | A-V | 1.00 |
|  | 1325 | TCA-TTA | S-L | 0.80 |
|  | 1432 | CTT-TTT | L-F | 0.80 |
| ndhF | 196 | CCT-TCT | P-S | 1.00 |
|  | 1867 | CTT-TTT | L-F | 1.00 |
|  | 1991 | ACA-ATA | T-I | 0.80 |
| ndhG | 314 | ACA-ATA | T-I | 0.80 |
|  | 494 | GCG-GTG | A-V | 0.80 |
| psaI | 83 | TCT-TTT | S-F | 0.86 |
| psbE | 214 | CCT-TCT | P-S | 1.00 |
| psbF | 77 | TCT-TTT | S-F | 1.00 |
| psbL | 2 | ACG-ATG | T-M | 1.00 |
| rpl2 | 1372 | CCC-TCC | P-S | 1.00 |
| rpoA | 821 | TCA-TTA | S-L | 1.00 |
| rpoB | 338 | TCT-TTT | S-F | 1.00 |
|  | 2426 | TCA-TTA | S-L | 0.86 |
| rpoC1 | 41 | TCA-TTA | S-L | 1.00 |
|  | 1562 | ACG-ATG | T-M | 0.86 |
| rpoC2 | 1505 | ACG-ATG | T-M | 0.86 |
|  | 1612 | CTT-TTT | L-F | 1.00 |
|  | 1807 | CGT-TGT | R-G | 0.86 |
|  | 1987 | CTT-TTT | S-L | 0.83 |
| rps2 | 248 | TCA-TTA | S-L | 1.00 |
| rps14 | 80 | TCA-TTA | S-L | 1.00 |
| rps16 | 212 | TCA-TTA | S-L | 0.83 |
| *Ficus simplicissima* | | | | |
| accD | 13 | CGG-TGG | R-W | 1.00 |
|  | 344 | CCA-CTA | P-L | 1.00 |
|  | 734 | TCT-TTT | S-F | 1.00 |
|  | 1421 | CCT-CTT | P-L | 1.00 |
| atpA | 773 | TCA-TTA | S-L | 1.00 |
|  | 791 | CCT-CTT | P-L | 1.00 |
|  | 914 | TCA-TTA | S-L | 1.00 |
| atpB | 37 | CCC-TCC | P-S | 1.00 |
| atpF | 19 | CCT-TCT | P-S | 0.86 |
|  | 92 | CCA-CTA | P-L | 0.86 |
| atpI | 76 | CTC-TTC | L-F | 0.86 |
| clpP | 559 | CAC-TAC | H-Y | 1.00 |
| matK | 52 | CTC-TTC | L-F | 1.00 |
|  | 640 | CAT-TAT | H-Y | 1.00 |
|  | 1187 | TCA-TTA | S-L | 0.86 |
| ndhA | 107 | CCT-CTT | P-L | 1.00 |
|  | 341 | TCA-TTA | S-L | 1.00 |
|  | 566 | TCA-TTA | S-L | 1.00 |
|  | 1073 | TCC-TTC | S-F | 1.00 |
| ndhB | 1682 | TCA-TTA | S-L | 1.00 |
|  | 2000 | CCA-CTA | P-L | 1.00 |
|  | 2075 | ACG-ATG | T-M | 1.00 |
|  | 2119 | CAT-TAT | H-Y | 1.00 |
|  | 2144 | TCA-TTA | S-L | 0.80 |
|  | 2270 | CCA-CTA | P-L | 1.00 |
|  | 2279 | TCT-TTT | S-F | 1.00 |
|  | 2363 | TCA-TTA | S-L | 1.00 |
|  | 2369 | TCA-TTA | S-L | 1.00 |
|  | 2645 | TCA-TTA | S-L | 1.00 |
|  | 2788 | CAT-TAT | H-Y | 1.00 |
|  | 3014 | CCA-CTA | P-L | 1.00 |
| ndhD | 29 | ACG-ATG | T-M | 1.00 |
|  | 340 | CGG-TGG | R-W | 0.80 |
|  | 410 | CCA-CTA | P-L | 1.00 |
|  | 626 | TCA-TTA | S-L | 1.00 |
|  | 701 | TCG-TTG | S-L | 1.00 |
|  | 905 | TCA-TTA | S-L | 11.00 |
|  | 914 | CCT-CTT | P-L | 1.00 |
|  | 1103 | GCT-GTT | A-V | 1.00 |
|  | 1325 | TCA-TTA | S-L | 0.80 |
|  | 1432 | CTT-TTT | L-F | 0.80 |
| ndhF | 196 | CCT-TCT | P-S | 1.00 |
|  | 1876 | CTT-TTT | L-F | 1.00 |
|  | 2000 | ACA-ATA | T-I | 0.80 |
| ndhG | 314 | ACA-ATA | T-I | 0.80 |
|  | 494 | GCG-GTG | A-V | 0.80 |
| psaI | 83 | TCT-TTT | S-F | 0.86 |
| psbE | 214 | CCT-TCT | P-S | 1.00 |
| psbF | 77 | TCT-TTT | S-F | 1.00 |
| psbL | 2 | ACG-ATG | T-M | 1.00 |
| rpl2 | 1372 | CCC-TCC | P-S | 1.00 |
| rpoA | 821 | TCA-TTA | S-L | 1.00 |
| rpoB | 338 | TCT-TTT | S-F | 1.00 |
|  | 2426 | TCA-TTA | S-L | 0.86 |
| rpoC1 | 41 | TCA-TTA | S-L | 1.00 |
|  | 1562 | ACG-ATG | T-M | 0.86 |
| rpoC2 | 1505 | ACG-ATG | T-M | 0.86 |
|  | 1612 | CTT-TTT | L-F | 1.00 |
|  | 1807 | CGT-TGT | R-G | 0.86 |
|  | 1987 | CTT-TTT | S-L | 0.83 |
|  | 3061 | CTT-TTT | L-F | 0.86 |
| rps2 | 248 | TCA-TTA | S-L | 1.00 |
| rps14 | 80 | TCA-TTA | S-L | 1.00 |
| rps16 | 212 | TCA-TTA | S-L | 0.83 |
| *Ficus tinctoria* | | | | |
| accD | 13 | CGG-TGG | R-W | 1.00 |
|  | 344 | CCA-CTA | P-L | 1.00 |
|  | 734 | TCT-TTT | S-F | 1.00 |
|  | 1421 | CCT-CTT | P-L | 1.00 |
| atpA | 773 | TCA-TTA | S-L | 1.00 |
|  | 791 | CCT-CTT | P-L | 1.00 |
|  | 914 | TCA-TTA | S-L | 1.00 |
| atpB | 37 | CCC-TCC | P-S | 1.00 |
| atpF | 19 | CCT-TCT | P-S | 0.86 |
|  | 92 | CCA-CTA | P-L | 0.86 |
| atpI | 76 | CTC-TTC | L-F | 0.86 |
| clpP | 281 | TCA-TTA | S-L | 0.86 |
|  | 569 | CCT-CTT | P-L | 0.86 |
| matK | 52 | CTC-TTC | L-F | 1.00 |
|  | 640 | CAT-TAT | H-Y | 1.00 |
|  | 1187 | TCA-TTA | S-L | 0.86 |
| ndhA | 107 | CCT-CTT | P-L | 1.00 |
|  | 341 | TCA-TTA | S-L | 1.00 |
|  | 566 | TCA-TTA | S-L | 1.00 |
|  | 1073 | TCC-TTC | S-F | 1.00 |
| ndhB | 1682 | TCA-TTA | S-L | 1.00 |
|  | 2000 | CCA-CTA | P-L | 1.00 |
|  | 2075 | ACG-ATG | T-M | 1.00 |
|  | 2119 | CAT-TAT | H-Y | 1.00 |
|  | 2144 | TCA-TTA | S-L | 0.80 |
|  | 2270 | CCA-CTA | P-L | 1.00 |
|  | 2279 | TCT-TTT | S-F | 1.00 |
|  | 2363 | TCA-TTA | S-L | 1.00 |
|  | 2369 | TCA-TTA | S-L | 1.00 |
|  | 2645 | TCA-TTA | S-L | 1.00 |
|  | 2788 | CAT-TAT | H-Y | 1.00 |
|  | 3014 | CCA-CTA | P-L | 1.00 |
| ndhD | 29 | ACG-ATG | T-M | 1.00 |
|  | 340 | CGG-TGG | R-W | 0.80 |
|  | 410 | CCA-CTA | P-L | 1.00 |
|  | 626 | TCA-TTA | S-L | 1.00 |
|  | 701 | TCG-TTG | S-L | 1.00 |
|  | 905 | TCA-TTA | S-L | 11.00 |
|  | 914 | CCT-CTT | P-L | 1.00 |
|  | 1103 | GCT-GTT | A-V | 1.00 |
|  | 1325 | TCA-TTA | S-L | 0.80 |
|  | 1432 | CTT-TTT | L-F | 0.80 |
| ndhF | 196 | CCT-TCT | P-S | 1.00 |
|  | 1876 | CTT-TTT | L-F | 1.00 |
|  | 2000 | ACA-ATA | T-I | 0.80 |
| ndhG | 314 | ACA-ATA | T-I | 0.80 |
|  | 494 | GCG-GTG | A-V | 0.80 |
| psaI | 83 | TCT-TTT | S-F | 0.86 |
| psbE | 214 | CCT-TCT | P-S | 1.00 |
| psbF | 77 | TCT-TTT | S-F | 1.00 |
| psbL | 2 | ACG-ATG | T-M | 1.00 |
| rpl2 | 1372 | CCC-TCC | P-S | 1.00 |
| rpoA | 821 | TCA-TTA | S-L | 1.00 |
| rpoB | 338 | TCT-TTT | S-F | 1.00 |
|  | 2426 | TCA-TTA | S-L | 0.86 |
| rpoC1 | 41 | TCA-TTA | S-L | 1.00 |
|  | 257 | TCT-TTT | S-F | 1.00 |
|  | 1562 | ACG-ATG | T-M | 0.86 |
| rpoC2 | 1505 | ACG-ATG | T-M | 0.86 |
|  | 1612 | CTT-TTT | L-F | 1.00 |
|  | 1807 | CGT-TGT | R-G | 0.86 |
|  | 1987 | CTT-TTT | S-L | 0.83 |
| rps2 | 248 | TCA-TTA | S-L | 1.00 |
| rps14 | 80 | TCA-TTA | S-L | 1.00 |
| rps16 | 212 | TCA-TTA | S-L | 0.83 |
