# Supplementary material for: Comprehensive analysis of complete chloroplast genome and phylogenetic aspects of ten Ficus species
Source: BMC Plant Biol. 2022 May 23;22:253. doi: 10.1186/s12870-022-03643-4 (PMC9125854; doi:10.1186/s12870-022-03643-4)
Supplement: Supplementary file 7 — Additional file 7: Table S6. Quality control of the Illumina sequencing of chloroplast genome of Ficus species. [file 12870_2022_3643_MOESM7_ESM.doc]

**Table S6**. Quality control of the Illumina sequencing of chloroplast genome of *Ficus* species

| sample_name | Clean_reads | **Clean**  bases | **Read**  length | Mean  coverage | Q20 | S**ingle-directio**n reads number | Single-direction bases number |
| --- | --- | --- | --- | --- | --- | --- | --- |
| *F.pumila* | 74752142 | 10877232784 | 150 bp | 1123.07 | 98.00% | 37376071 | 5438616392 |
| *F.tikoua* | 43837288 | 6235418668 | 150 bp | 2293.42 | 98.00% | 21918644 | 3117709334 |
| *F.hispida* | 61917344 | 8936087192 | 150 bp | 852.44 | 98.00% | 30958672 | 4468043596 |
| *F.virens* | 67305312 | 9764642402 | 150 bp | 815.72 | 98.00% | 33652656 | 4882321201 |
| *F.sarmentosa var. impressa* | 67173738 | 9790810870 | 150 bp | 827.17 | 98.00% | 33586869 | 4895405435 |
| *F.sarmentosa var. lacrymans* | 58641742 | 8230358726 | 150 bp | 944.16 | 98.00% | 29320871 | 4115179363 |
| *F.pandurata* | 40789588 | 5807658496 | 150 bp | 1081.24 | 98.00% | 20394794 | 2903829248 |
| *F.tinctoria* | 62221630 | 8792750456 | 150 bp | 537.88 | 98.00% | 31110815 | 4396375228 |
| *F.formosana* | 52237124 | 7483036466 | 150 bp | 1563.67 | 98.00% | 26118562 | 3741518233 |
| *F.microcarpa* | 65770852 | 9546628798 | 150 bp | 703.86 | 98.00% | 32885426 | 4773314399 |
| *F.simplicissima* | 43946964 | 6274707632 | 150 bp | 829.01 | 98.00% | 21973482 | 3137353816 |
