# Supplementary material for: Comprehensive analysis of complete chloroplast genome and phylogenetic aspects of ten Ficus species
Source: BMC Plant Biol. 2022 May 23;22:253. doi: 10.1186/s12870-022-03643-4 (PMC9125854; doi:10.1186/s12870-022-03643-4)
Supplement: Supplementary file 8 — Additional file 8: Table S7. Universal primers for amplifying four regions between the IR regions and the LSC/SSC region. [file 12870_2022_3643_MOESM8_ESM.doc]

**Table S7.** Universal primers for amplifying four regions between the IR regions and the LSC/SSC region

| Species | LSC/IRB region | IRB/SSC region | SSC/IRA region | IRA/LSC region |
| --- | --- | --- | --- | --- |
| *Ficus pumila* | Forward primer (5'-3') GGCAAATGTTCCTTTCCATT  Reverse primer (5'-3')  AAAAATCCTTTTGTAGCCAATCA | Forward primer (5'-3') CCGTTTATGAAACTGATTATCTGG  Reverse primer (5'-3')  GGTGTGGGTCGAATCTCTTC | Forward primer (5'-3') CAGCATTTTCAAATCGATTCTTT  Reverse primer (5'-3')  TCTGGATATGGATCGGAATCA | Forward primer (5'-3') TTGTGGCACGTTCACTAAAA  Reverse primer (5'-3')  CGTCGTTCGCCCATTTATTA |
| *Ficus tikoua* | Forward primer (5'-3') AGCGATAGTATGCCCGATCA  Reverse primer (5'-3')  TTGTGGCACGTTCACTAAAAA | Forward primer (5'-3') CCGTTTATGAAACTGATTATCTGG  Reverse primer (5'-3')  GGTGTGGGTCGAATCTCTTC | Forward primer (5'-3') CAGCATTTTCAAATCGATTCTTT  Reverse primer (5'-3')  TCTGGATATGGATCGGAATCA | Forward primer (5'-3') ACGTTCACTAAAAAAAAATCCTTTT  Reverse primer (5'-3')  GTTACTATTATTTCTTTTTCCGCTT |
| *Ficus hispida* | Forward primer (5'-3') GTGCAAATTCCCCCAACTTA  Reverse primer (5'-3')  GCTTAACACAAAAGCGGAAAA | Forward primer (5'-3') TGAATTCCACTTTAAAGAGACACG  Reverse primer (5'-3')  TGTGGGTCGAATCTCTTCCT | Forward primer (5'-3') CAGCATTTTCAAATCGATTCTTT  Reverse primer (5'-3')  TCTGGATATGGATCGGAATCA | Forward primer (5'-3') TTGTGGCACGTTCACTAAAA  Reverse primer (5'-3')  CGTCGTTCGCCCATTTATTA |
| *Ficus virens* | Forward primer (5'-3') TCCCCCAACTTATGGCCTAC  Reverse primer (5'-3')  GCTTAACACAAAAGCGGAAAA | Forward primer (5'-3') TGAATTCCACTTTAAAGAGACACG  Reverse primer (5'-3')  TGTGGGTCGAATCTCTTCCT | Forward primer (5'-3') CAGCATTTTCAAATCGATTCTTT  Reverse primer (5'-3')  TCTGGATATGGATCGGAATCA | Forward primer (5'-3') TTAATTGTGGCACGTTCACTAAAAA  Reverse primer (5'-3')  ACTATTATCTCTTTTTCCGCTTTTG |
| *Ficus sarmentosa var. impressa* | Forward primer (5'-3') CCCCAACTTATGGCCTACCATACGAReverse primer (5'-3')  AAAATCCTTTTGTAGCCAATCATTT | Forward primer (5'-3') CCGTTTATGAAACTGATTATCTGG  Reverse primer (5'-3')  GGTGTGGGTCGAATCTCTTC | Forward primer (5'-3') CAGCATTTTCAAATCGATTCTTT  Reverse primer (5'-3')  TCTGGATATGGATCGGAATCA | Forward primer (5'-3') GGCAAATGTTCCTTTCCATT  Reverse primer (5'-3')  GTGGATCAAGGCAGTGGATTGTGAA |
| *Ficus sarmentosa var. lacrymans* | Forward primer (5'-3') TTCCCCCAACTTATGGCCTACCATA  Reverse primer (5'-3')  CACGTTCACTAAAAAAAAATCCTTT | Forward primer (5'-3') TGAATTCCACTTTAAAGAGACACG  Reverse primer (5'-3')  TGTGGGTCGAATCTCTTCCT | Forward primer (5'-3') CAGCATTTTCAAATCGATTCTTT  Reverse primer (5'-3')  TCTGGATATGGATCGGAATCA | Forward primer (5'-3') TTGTGGCACGTTCACTAAAAA  Reverse primer (5'-3')  GTGGATCAAGGCAGTGGATT |
| *Ficus pandurata* | Forward primer (5'-3') ATTCCCCCAACTTATGGCCTACCAT  Reverse primer (5'-3')  TGGCACGTTCACTAAAAAAAAATCC | Forward primer (5'-3') CCGTTTATGAAACTGATTATCTGG  Reverse primer (5'-3')  GGTGTGGGTCGAATCTCTTC | Forward primer (5'-3') CAGCATTTTCAAATCGATTCTTT  Reverse primer (5'-3')  TCTGGATATGGATCGGAATCA | Forward primer (5'-3') ATTAATTGTGGCACGTTCACTAAAA Reverse primer (5'-3')  TAGCCAAGTGGATCAAGGCAGTGGA |
| *Ficus tinctoria* | Forward primer (5'-3') GCAAATTCCCCCAACTTATG  Reverse primer (5'-3')  GCTTAACACAAAAGCGGAAAA | Forward primer (5'-3') TGAATTCCACTTTAAAGAGACACG  Reverse primer (5'-3')  TGTGGGTCGAATCTCTTCCT | Forward primer (5'-3') CAGCATTTTCAAATCGATTCTTT  Reverse primer (5'-3')  TCTGGATATGGATCGGAATCA | Forward primer (5'-3') TTGTGGCACGTTCACTAAAAA  Reverse primer (5'-3')  GTGGATCAAGGCAGTGGATT |
| *Ficus formosana* | Forward primer (5'-3') GTGCAAATTCCCCCAACTTA  Reverse primer (5'-3')  GCTTAACACAAAAGCGGAAAA | Forward primer (5'-3') TCTGGATATGGATCGGAATCA  Reverse primer (5'-3')  GGGGTCGGTATTTCGAGTTT | Forward primer (5'-3') CAGCATTTTCAAATCGATTCTTT  Reverse primer (5'-3')  TCTGGATATGGATCGGAATCA | Forward primer (5'-3')  GCTTAACACAAAAGCGGAAAA  Reverse primer (5'-3')  TCAAGGCAGTGGATTGTGAA |
| *Ficus microcarpa* | Forward primer (5'-3') GTGCAAATTCCCCCAACTTA  Reverse primer (5'-3')  GCTTAACACAAAAGCGGAAAA | Forward primer (5'-3') TGAATTCCACTTTAAAGAGACACG  Reverse primer (5'-3')  TGTGGGTCGAATCTCTTCCT | Forward primer (5'-3') CAGCATTTTCAAATCGATTCTTT  Reverse primer (5'-3')  TCTGGATATGGATCGGAATCA | Forward primer (5'-3') AAAATAGGAGTAATTAATTGTGGCA  Reverse primer (5'-3')  CAAGTGGATCAAGGCAGTGGATTGT |
| *Ficus simplicissima* | Forward primer (5'-3') GTGCAAATTCCCCCAATTTA  Reverse primer (5'-3')  GCTTAACACAAAAGCGGAAAA | Forward primer (5'-3') TCTGGATATGGATCGGAATCA  Reverse primer (5'-3')  GGGTCGGTATTTCGAGTTTTT | Forward primer (5'-3') CAGCATTTTCAAATCGATTCTTT  Reverse primer (5'-3')  TCTGGATATGGATCGGAATCA | Forward primer (5'-3') GTGGCACGTTCACTAAAAAAAAATC  Reverse primer (5'-3')  GGCAGTGGATTGTGAATCCACCATG |
