# Supplementary material for: Comprehensive analysis of complete chloroplast genome and phylogenetic aspects of ten Ficus species
Source: BMC Plant Biol. 2022 May 23;22:253. doi: 10.1186/s12870-022-03643-4 (PMC9125854; doi:10.1186/s12870-022-03643-4)
Supplement: Supplementary file 9 — Additional file 9: Table S8. The 23 studied species and the corresponding chloroplast whole genome GenBank accession number. [file 12870_2022_3643_MOESM9_ESM.doc]

**Table S8**. The 23 studied species and the corresponding chloroplast whole genome GenBank accession number

| Genus | Species | GenBank No. |
| --- | --- | --- |
| *Ficus* | *F. erecta* | MT093220 |
|  | *F. beipeiensis* | MH045575 |
|  | *F. racemosa* | NC_028185 |
|  | *F. religiosa* | NC_033979 |
|  | *F. carica* | NC_035237 |
|  | *F. hirta* | MN364706 |
| *Morus* | *M. notabilis* | NC_027110 |
|  | *M. cathayana* | NC_031822 |
|  | *M. multicaulis* | KU355297 |
|  | *M. mongolica* | KM491711 |
|  | *M. celtidifolia* | NC_047236 |
|  | *M.. alba* | KU355276 |
|  | *M. indica* | DQ226511 |
| *Malaisia* | *Malaisia scandens* | MH189568 |
| *Broussonetia* | *B. luzonica* | NC_047180 |
|  | *B. kazinoki* | MH223642 |
|  | *B.papyrifera* | MH189570 |
|  | *B. kurzii* | NC_041637 |
|  | *B.kaempferi* | NC_047183 |
|  | *B. monoica* | NC_047181 |
| *Artocarpus* | *A. heterophyllus* | MK303549 |
| *Cannabis* | *Cannabis sativa* | NC_026562 |
| *Rhamnus* | *R. taquetii* | NC_045855 |
